# Simultaneous activation of Tor and suppression of ribosome biogenesis by TRIM-NHL proteins promotes terminal differentiation

**DOI:** 10.1016/j.celrep.2023.112181

**Published:** 2023-03-03

**Authors:** Jinghua Gui, Tamsin J. Samuels, Katarina Z.A. Grobicki, Felipe Karam Teixeira

**Affiliations:** 1 Department of Genetics, University of Cambridge, Downing Street, Cambridge CB2 3EH, UK; 2 Department of Physiology, Development and Neuroscience, University of Cambridge, Downing Street, Cambridge CB2 3DY, UK

## Abstract

Tissue development and homeostasis depend on the balance between growth and terminal differentiation, but the mechanisms coordinating these processes remain elusive. Accumulating evidence indicates that ribosome biogenesis (RiBi) and protein synthesis, two cellular processes sustaining growth, are tightly regulated and yet can be uncoupled during stem cell differentiation. Using the *Drosophila* adult female germline stem cell and larval neuroblast systems, we show that Mei-P26 and Brat, two *Drosophila* TRIM-NHL paralogs, are responsible for uncoupling RiBi and protein synthesis during differentiation. In differentiating cells, Mei-P26 and Brat activate the target of rapamycin (Tor) kinase to promote translation, while concomitantly repressing RiBi. Depletion of Mei-P26 or Brat results in defective terminal differentiation, which can be rescued by ectopic activation of Tor together with suppression of RiBi. Our results indicate that uncoupling RiBi and translation activities by TRIM-NHL activity creates the conditions required for terminal differentiation.

## Graphical abstract

**Graphical abstract:**
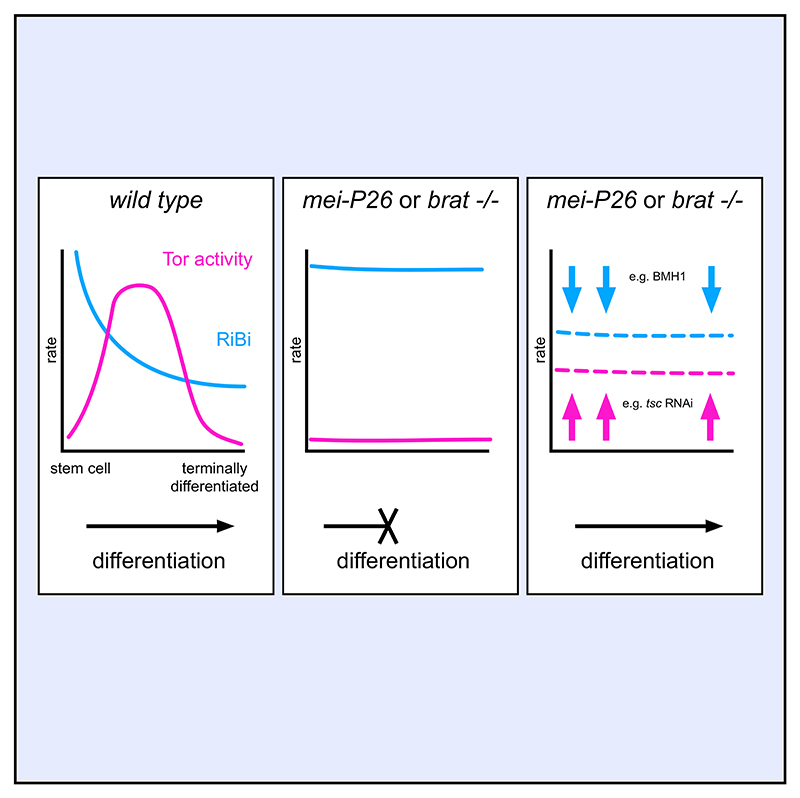

## Introduction

The tight coordination of cellular metabolic activities is essential for cell growth, proliferation, stress response, and cell survival, and therefore is at the core of the developmental processes sculpting complex organs and enabling robust tissue repair and homeostasis in adults.^1–3^ For instance, translation and ribosome biogenesis (RiBi), two of the most energy-consuming anabolic activities, have been shown to be dynamically regulated during cell fate transitions, with stem cells differing significantly from their immediate differentiating progeny.^4^ This dynamic regulation has been observed in many stem cell systems, including the *Drosophila* adult germline stem cells (GSCs), intestinal stem cells, and larval neuroblasts (NBs),^5–9^ as well as mouse adult stem cells in the hematopoietic, neural, muscle, and skin systems.^10–15^ Genetic and pharmacological manipulations revealed that the control of these metabolic activities is critically important for tissue homeostasis, tipping the balance between self-renewal and differentiation.^1,8,10,11,14^ However, even though dynamic changes in translation and RiBi are required during stem cell differentiation and are pervasive across different systems, the mechanisms by which they are regulated remain poorly understood.

The *Drosophila* ovary presents an ideal *in vivo* system for dissecting the regulation of metabolism during stem cell differentiation.^16^ GSCs, found attached to the somatic niche at the anterior most part of the ovaries show lower translational activity and higher RiBi rates in comparison with neighboring differentiating cells.^7–9^ Upon stem cell division and niche exclusion, the differentiating cystoblast (CB) undergoes four rounds of mitosis with incomplete cytokinesis before terminally differentiating as a 16-cell cyst. The differentiating cyst stages are characterized by higher translation and lower RiBi compared with the stem cell (Figure S1A,^8^), which is thought to result in limited production of new ribosomes alongside high demand for protein synthesis. Translation and RiBi are usually closely coordinated to ensure that adequate numbers of ribosomes are available to sustain the required levels of protein synthesis.^17^ Therefore, it is not surprising that the metabolic changes observed during GSC differentiation are associated with changes in growth, with differentiating progeny decreasing in cell size prior to terminal differentiation.^7^ Notably, experimental modulation of RiBi and protein synthesis activities during germline differentiation has been shown to affect the balance between self-renewal and differentiation, resulting in either premature loss of GSCs, a block in differentiation, or tumorigenesis.^7–9,18^

A known regulator of RiBi during GSC differentiation is meiotic P26 (Mei-P26), a germline-expressed gene encoding a member of the evolutionarily conserved TRIM-NHL family of proteins (the mammalian TRIM family).^7,19^ In *mei-P26* mutant ovaries, differentiating cysts maintain high RiBi rates that are usually characteristic of stem cells, overgrow, and fail to terminally differentiate, leading to the formation of tumors composed of partially differentiated cysts.^7,19^ Similarly, in the *Drosophila* larval brain, mutants of the *mei-P26* paralog *brain tumor* (*brat*) are characterized by the failure of differentiation of the progeny of the larval NBs. *brat* mutant progeny cells show increased RiBi rates, large cell size, and excessive proliferation—leading to a characteristic brain tumor phenotype.^5,20^

Here, we delve into the mechanisms that control protein synthesis and RiBi activity during stem cell differentiation. First, we show that the activity of the target of rapamycin (Tor) kinase—an evolutionarily conserved regulator of cell metabolism that generally coordinates translation and RiBi to drive growth—is developmentally regulated during stem cell differentiation. We demonstrate that Tor activation drives the observed increase in protein synthesis during germline differentiation. Surprisingly, we find that Mei-P26 and Brat are activators of the Tor kinase, alongside their previously identified roles in suppressing RiBi. While *mei-P26* and *brat* mutant cells do not activate the Tor kinase during differentiation, overexpression of these TRIM-NHL proteins leads to ectopic Tor activation, resulting in premature differentiation. Using genetic and pharmacological manipulations, we show that the *mei-P26-* or *brat-*induced block in differentiation can be resolved by restoring RiBi suppression and Tor activation, revealing that the metabolic uncoupling driven by TRIM-NHL proteins is critical for inducing terminal differentiation.

## Results

### Tor is activated during GSC differentiation and is required for the increase in translation rate

We have previously shown that RiBi and protein synthesis rates are actively regulated and yet uncoupled during GSC differentiation: RiBi must be enhanced in the GSCs to allow correct initiation of differentiation, then during the differentiation process protein synthesis increases alongside a reduction in RiBi.^8^ This un-coupling is at odds with the well-established role of the evolutionarily conserved Tor kinase in coordinating RiBi and protein synthesis activities to promote growth.^17^ Tor kinase activity has previously been shown to be involved in GSC proliferation and cyst growth,^8,18,21^ but the loss of Tor activity does not affect RiBi in GSCs,^8^ raising the question of when the Tor kinase is active during GSC differentiation. To investigate this, we took advantage of an antibody against the phosphorylated form of the ribosomal protein S6 (p-S6), a downstream target and readout of the activity of the Tor pathway.^22,23^

**Figure 1:**
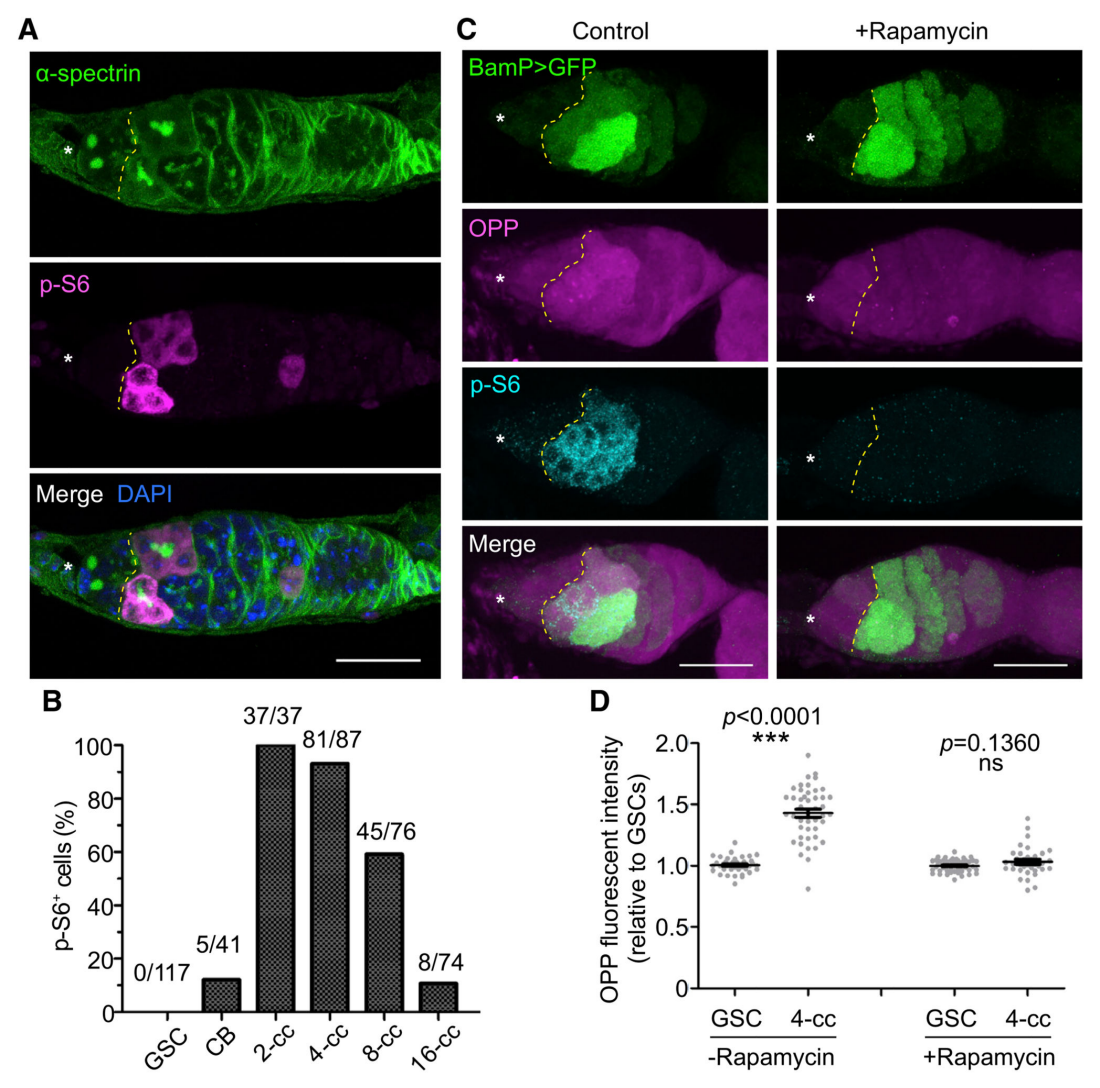
Tor is activated during germline stem cell differentiation and drives the observed increase in translation (A) A wild-type germarium labeled with α-spectrin (spectrosomes/fusomes, green), p-S6 (Tor activity, magenta), and DAPI (nuclei, blue). (B) Quantitation of the proportion of p-S6^+^ cells at different stages of germline differentiation. (C) Germaria with or without 10 μM rapamycin treatment, expressing the differentiation marker BamP>GFP (green), labeled with OPP (translation rate, magenta), and p-S6 (cyan). (D) OPP fluorescent intensity measurements of germaria with or without rapamycin treatment. Data are mean ± SEM. ***p < 0.0001, t test. Asterisks indicate the GSC niche. Dashed lines indicate the boundary between GSCs and differentiating cells (A and C). Scale bars, 20 μm (A and C).

Immunofluorescence microscopy analysis revealed that GSCs were devoid of p-S6, but a strong p-S6 signal was detectable from the differentiating CB stage onward (Figures 1A, 1B, and S1A). While only ~12% of CBs were positively marked by p-S6, all 2-cell and ~93% of 4-cell cysts were p-S6^+^, with the penetrance of p-S6 signal declining in 8-cell (~59%) and terminally differentiated 16-cell cysts (~11%). Analysis using the cell-cycle tracing FUCCI system^24^ confirmed that p-S6 expression was independent of cell-cycle phase (Figures S1B–S1D). Moreover, a short incubation of adult ovaries with rapamycin, a specific inhibitor of the Tor kinase, was sufficient to abolish the p-S6 signal in differentiating cells (Figure 1C). As the Tor kinase and the target of rapamycin complex 1 (TORC1) co-factor Raptor proteins are present from GSCs to 16-cell cysts (Figure S1E), our results indicate that Tor is inactive in the GSCs but is activated during differentiation.

Differentiating germ cells are characterized by a significant increase in protein synthesis rate,^8^ which temporally coincides with our observation of Tor activation. To test whether Tor mediates the increase in translation, we measured global protein synthesis rates *in vivo* using an imaging-based assay for O-propargyl-puromycin (OPP) incorporation into nascent polypeptides, which serves as a proxy for translation output.^8,25^ The robust increase in OPP incorporation observed in control differentiating cells was abolished by a short incubation with rapamycin (Figures 1C and 1D). These results demonstrate that Tor is developmentally activated during cyst differentiation and plays a major role in promoting the observed increase in translation.

### Tor activation during GSC differentiation depends on the amino acid sensing pathway, not the insulin pathway

**Figure 2:**
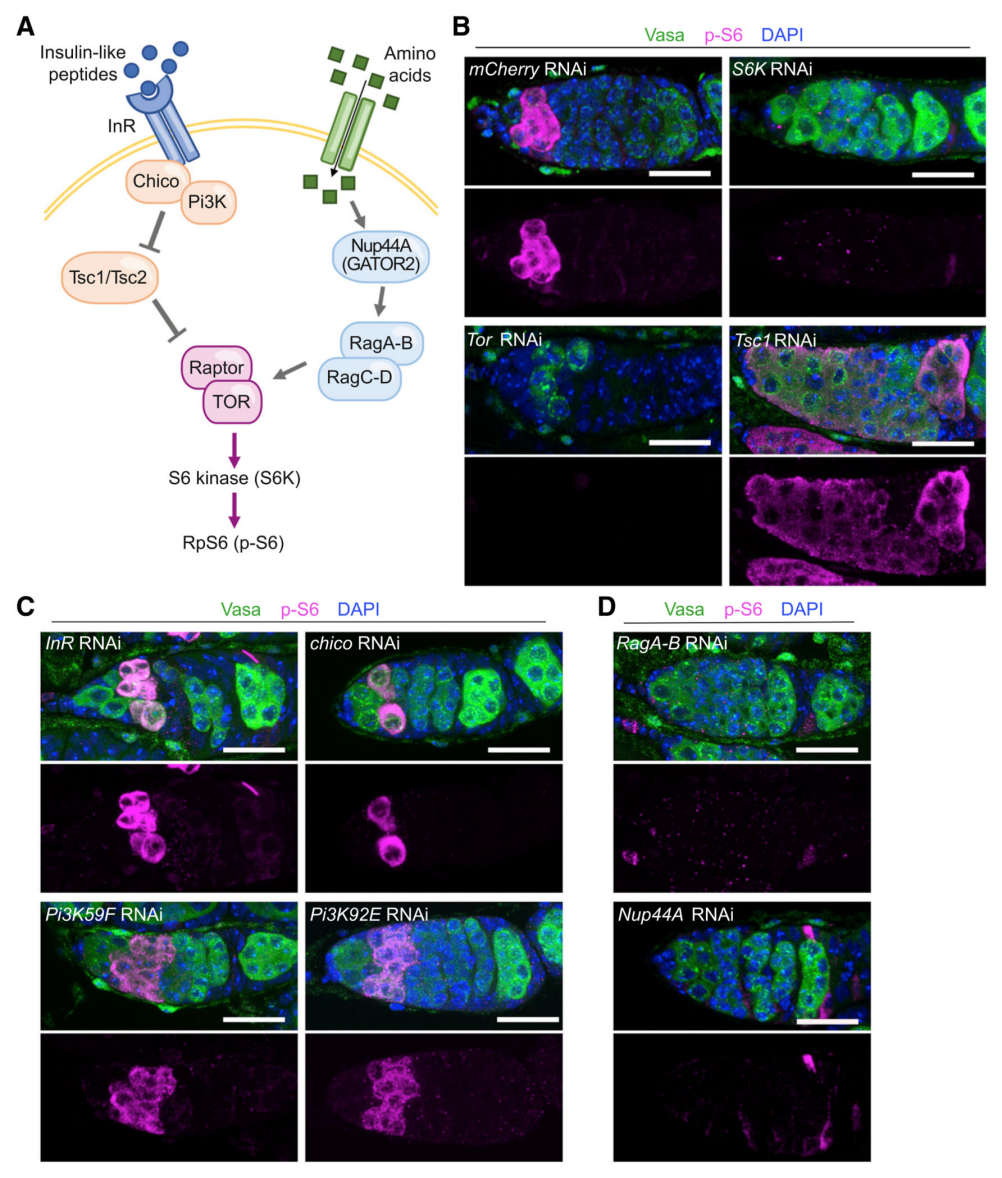
RagAB and Nup44A are required for Tor activation during GSC differentiation (A) A simplified schematic showing insulin and amino acid sensing pathways upstream of the Tor kinase, depicting the genes that were tested here. (B) Germaria of germline-specific KD of control (*mCherry* RNAi), *Tor, S6K*, or *tsc1* labeled with Vasa (germline marker, green), p-S6 (magenta and individual channel), and DAPI (blue). (C and D) Germaria of germline-specific KD of *InR, chico, Pi3K59F (Vps34*), *Pi3K92E (Dp110*), *RagAB*, or *Nup44A* labeled with Vasa (green), p-S6 (magenta), and DAPI (blue). Scale bars, 20 μm (B–D).

To determine which of the upstream molecular pathways participate in Tor kinase activation during GSC differentiation, we took advantage of tissue-specific RNAi knockdown (KD) of key pathway components (Figure 2A). As expected, the p-S6 signal was abolished upon KD of either the Tor kinase itself or the downstream effector kinase ribosomal protein S6 kinase (S6K) (Figure 2B). In addition, KD of Tsc1, a direct inhibitor of Tor kinase activity,^26,27^ resulted in a low, uniform p-S6 expression throughout the germarium, including the GSCs (Figure 2B).

The insulin receptor (InR)/phosphoino-sitide 3-kinase (PI3K)/AKT signaling cascade is one of the most established upstream activators of the Tor kinase during cell growth (Figure 2A). However, p-S6 expression was minimally affected upon KD of key components of the InR/PI3K cascade, including InR, chico/InR receptor substrate, and the PI3K catalytic subunits Dp110/Pi3K92E and Vps34/Pi3K59F (Figure 2C). These data indicate that the InR/PI3K cascade is not a major activator of the Tor kinase during GSC differentiation, in agreement with the previous observation that the null *InR* mutant had a much less severe cyst growth delay than the null *Tor* mutant.^21^ A second established upstream pathway regulating Tor depends on amino acid sensing, which can activate the Tor kinase through the Rag GTPases RagAB and RagCD (Figure 2A). When knocking down RagAB in germ cells, we observed that the p-S6 expression during GSC differentiation was abolished, similar to what was observed when knocking down Tor or S6K (Figure 2D). Further upstream in the amino acid sensing pathway, GATOR2 (Seh1-associated complex activates TORC1, or SEACAT, in yeast) activates Tor through the inhibition of the inhibitory GATOR1 complex.^28^ KD of the GATOR2 component Nup44A/seh1 also resulted in loss of p-S6 expression during differentiation (Figures 2A and 2D). These findings suggest that the amino acid sensing module is upstream of Tor activation during GSC differentiation, rather than the InR/PI3K pathway.

### Mei-P26 activates Tor during GSC differentiation, uncoupling protein synthesis and RiBi

**Figure 3:**
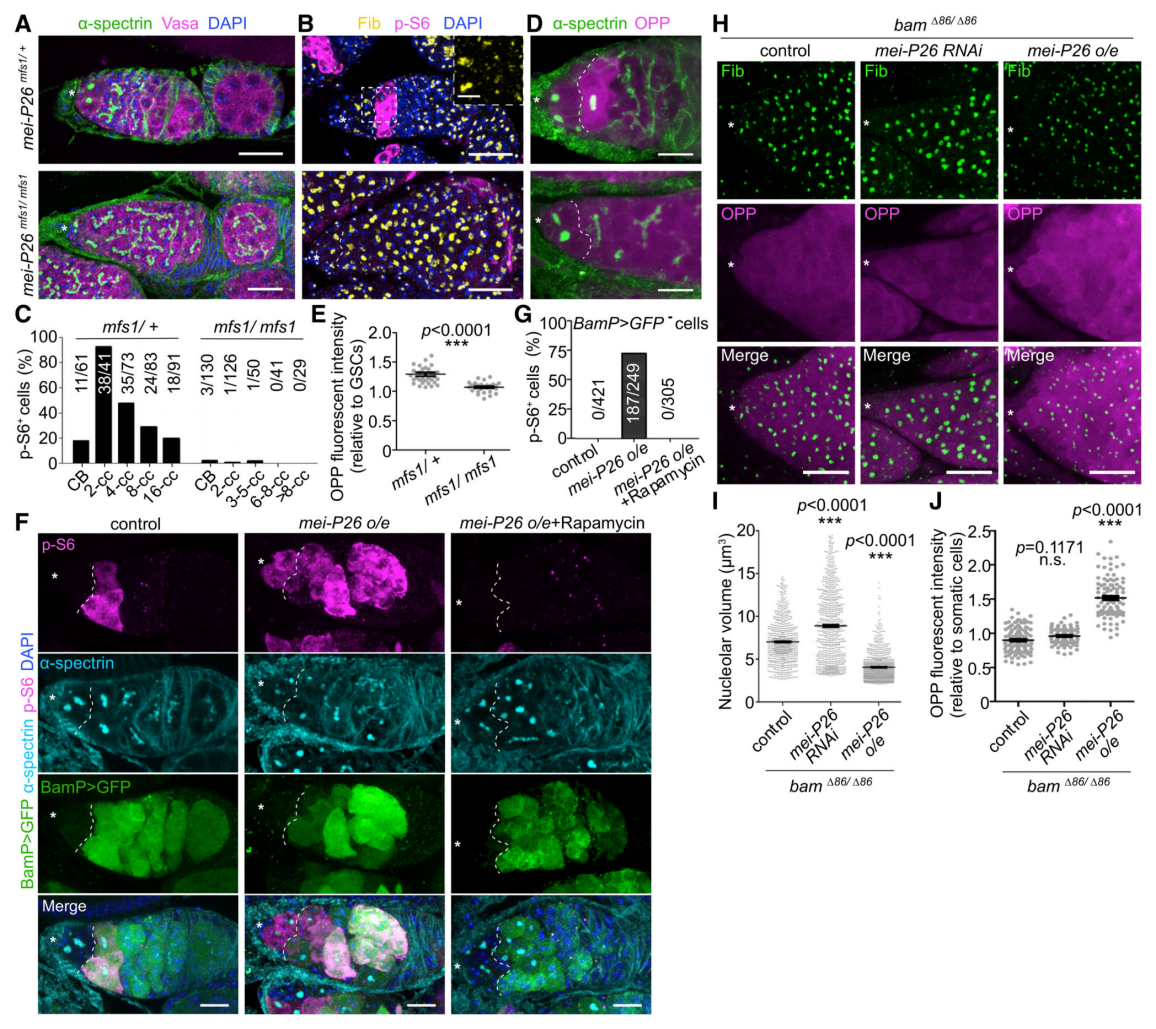
Mei-P26 activates Tor kinase during GSC differentiation (A, B, and D) Germaria of *mei-P26*^*mfs1/+*^ and *mei-P26*^*mfs1/mfs1*^ flies stained with α-spectrin (spectrosomes/fusomes, green, A and D), Vasa (magenta, A), DAPI (blue, A and B), Fib (nucleoli, yellow, B), p-S6 (magenta, B), and OPP (magenta, D). (C) Quantitation of the proportion of p-S6^+^ cells at different stages of germline differentiation from *mei-P26*^*mfs1/+*^ and *mei-P26*^*mfs1/mfs1*^ flies. (E) OPP fluorescent intensity measurements of two- to four-cell cysts (cc) relative to GSCs, from germaria of *mei-P26*^*mfs1/+*^ or *mei-P26*^*mfs1/mfs1*^ flies. (F) Germaria of control (*nos-gal4/+*) and mei-P26 overexpression (o/e) (*nos-gal4/UASp-mei-P26*) flies with or without rapamycin feeding, stained with p-S6 (magenta), α-spectrin (cyan), GFP (BamP>GFP, differentiating cells, green) and DAPI (blue). (G) Proportion of p-S6^+^ GSCs in germaria of control (*nos-gal4/+*) and Mei-P26 o/e (*nos-gal4/UASp-mei-P26*) with or without rapamycin feeding. (H) Germaria of control (*nos-gal4/+*), *mei-P26* KD (*nos-gal4/UAS-mei-P26 RNAi*), or *mei-P26* o/e (*nos-gal4/UASp-mei-P26*) in the *bam*^*Δ*86/*Δ*86^ background, stained with Fib (green) and OPP (magenta). (I and J) Measurements of nucleolar volume per cell (I) and translation rates (J) in GSC-like cells (*bam*^*Δ*86^) in germaria of *mei-P26* KD (*nos-gal4/UAS-mei-P26 RNAi*) or *mei-P26* o/e (*nos-gal4/UASp-mei-P26*) in the *bam*^*Δ*86/*Δ*86^ background. Data are mean ± SEM. ***p < 0.0001, t test (E, I, and J). Asterisks indicate the GSC niche. Dashed lines indicate the boundary between GSCs and differentiated cells (A, B, D, and F). Scale bars, 20 μm (A, B, F, and H), 10 μm (D), or 5 μm (B, inset).

Mei-P26, a TRIM-NHL protein ortholog of the mammalian TRIM family of proteins, has been reported to negatively regulate nucleolar size (a proxy for RiBi) during GSC differentiation.^7^ Phenotypically, *mei-P26*^*mfs1/mfs1*^ mutants initiate the GSC differentiation program, but differentiating cysts show enlarged nucleoli, increased cellular volume, and are unable to complete terminal differentiation (Figures 3A and 3B).^7^ Surprisingly, our analysis revealed that differentiating cysts in *mei-P26*^*mfs1/mfs1*^ mutants were also devoid of p-S6 signal, suggesting that Mei-P26 is required for Tor activation during differentiation (Figures 3B and 3C). Furthermore, the increase in global translation rate that is observed in differentiating cysts was abolished in *mei-P26*^*mfs1/mfs1*^ mutants (Figures 3D and 3E).

To determine whether Tor kinase activity is directly promoted by Mei-P26, we overexpressed Mei-P26 in wild-type germ cells, which resulted in >75% (187 out of 249) of GSCs showing ectopic p-S6 signal (Figures 3F and 3G). In addition, extensive p-S6 signal was detected in fully differentiated egg chambers in *mei-P26* overexpressing (OE) ovaries (Figure S2A). It has been shown that sustained *mei-P26* OE induces premature GSC differentiation, eventually leading to loss of germ cells.^7^ We found that feeding flies with rapamycin not only abolished p-S6 signal in *mei-P26* OE ovaries but was also sufficient to suppress the GSC loss phenotype (Figures 3F, 3G, S2A, and S2B), indicating that ectopic Tor activation underlies Mei-P26-dependent premature loss of GSCs.

To characterize the molecular effects of modulating Mei-P26 expression in GSCs, we coupled overexpression and KD of Mei-P26 with a loss-of-function mutation for the differentiation factor Bag-of-marbles (Bam),^29^ which completely blocks GSC differentiation (Figure S2C). Immunofluorescence analyses revealed that OPP incorporation was positively correlated with Mei-P26 levels in *bam*^*Δ*86^ GSC-like cells while confirming that nucleolar volume displays an inverse correlation with Mei-P26 expression (Figures 3H–3J).^7^ Indeed, RNA sequencing experiments on these *bam*^*Δ*86^ GSC-like cells with either *mei-P26* KD or OE revealed that RiBi-related genes—including those encoding ribosomal proteins, nucleolar markers, and ribosomal RNA (rRNA) polymerases—were negatively regulated by Mei-P26 (Figure S2D; Table S1). Our results demonstrate that Mei-P26 activates Tor to promote increased translation and, together with the previously shown role of Mei-P26 to suppress RiBi, this uncouples two of the most important anabolic processes during germline differentiation.

### Both the TRIM motif and NHL repeats are necessary for Mei-P26 to activate Tor

**Figure 4:**
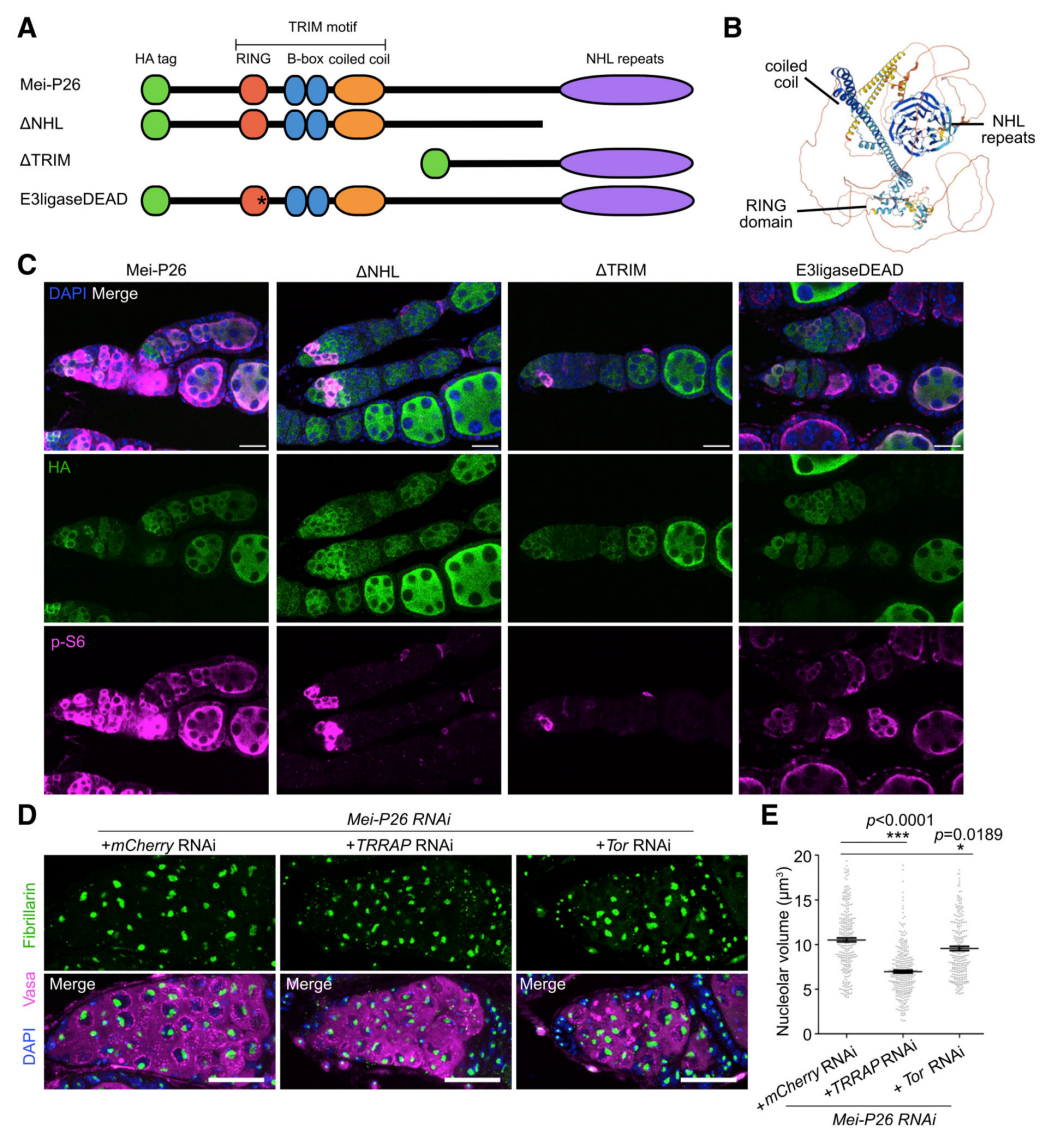
Mei-P26 activates Tor through its NHL and TRIM domains, and suppresses RiBi via TRRAP/Nipped-A (A) Transgenic constructs were generated using a UASp promoter to overexpress either the full-length Mei-P26, deletion of the NHL repeats (ΔNHL), deletion of the N-terminal region including the TRIM motif (ΔTRIM), or a point mutation rendering the E3 ligase nonfunctional (E3ligase-DEAD). All constructs were HA tagged. (B) The AlphaFold prediction for Mei-P26 structure, showing the β propellor formed by the NHL repeats.^32,34^ (C) Ovarioles stained for HA (green), p-S6 (magenta) and DAPI (blue) for each over-expression construct, driven by nanos-GAL4. (D) Germaria of germline-specific KD of *mei-P26* together with KD of *mCherry, TRRAP/Nipped-A*, or *Tor*, labeled with Fib (green), Vasa (magenta), and DAPI (blue). (E) Measurements of nucleolar volume per cell in (D). Data are mean ± SEM. ***p < 0.0001, t test (E). Scale bars, 20 μm.

To distinguish the roles of the Mei-P26 protein domains in activating Tor during GSC differentiation, we produced transgenic flies containing inducible constructs (Figure 4A). Mei-P26 contains an N-terminal TRIM motif (consisting of a RING domain, two B-box zinc fingers, and a coiled coil) and C-terminal NHL repeats that form a β propellor structure (Figure 4B).^30–34^ We generated HA-tagged constructs (Figure 4A) to overexpress the full Mei-P26 protein (Mei-P26), a Mei-P26 with a deletion of the NHL repeats (ΔNHL), a deletion of the N-terminal region including the TRIM motif (ΔTRIM), and the full-length Mei-P26 with a point mutation rendering the RING domain E3 ubiquitin ligase dead (E3ligaseDEAD). We expressed these different constructs with a nanos-GAL4 driver and stained the ovaries for HA and p-S6 to examine Tor activation (Figure 4C). As shown in Figure 3F, we found that overexpression of Mei-P26 resulted in expansion of the p-S6-positive domain to include the stem cells and egg chambers. However, the deletions of the NHL repeats or TRIM motif both resembled wild-type p-S6 expression, with no ectopic p-S6 signal. Overexpression of the E3liga-seDEAD construct was indistinguishable from the intact Mei-P26 overexpression, suggesting that the E3 ubiquitin ligase activity is not required for Mei-P26 activation of Tor. We conclude that both the TRIM motif and the NHL repeats are individually required for regulation of the Tor kinase by Mei-P26.

### Mei-P26 regulates RiBi through the pseudokinase TRRAP

It was previously shown that high levels of RiBi in the GSCs are sustained mainly by TRRAP.^8^ TRRAP, encoded by Nipped-A in *Drosophila*, is the only pseudokinase in the phosphatidylinositol 3-kinase-related kinase (PIKK) protein family, which also includes Tor, ATM, and ATR.^35^ While TRRAP is required for high RiBi in the GSCs, its role during differentiation is unclear. To test whether the high rate of RiBi observed in differentiating *mei-P26* mutant cysts depends on TRRAP, we measured nucleolar size in *TRRAP* KD in the background of *mei-P26* KD. Enlarged nucleolar size in differentiating cysts in the *mei-P26* KD was significantly reduced upon additional *TRRAP* KD, compared with control *mCherry* KD (Figures 4D and 4E). As expected, KD of the Tor kinase only marginally affected the nucleolar size in the *mei-P26* KD (Figures 4D and 4E). This is in agreement with our observation that Tor activity is lost in the *mei-P26* KD, as well as the previous finding that Tor has a minimal role in the high RiBi rates observed in GSCs.^8^ Altogether, our data suggest that Mei-P26 regulates two PIKKs during differentiation, promoting translation through the activation of Tor while decreasing RiBi through TRRAP.

### Redressing RiBi and Tor activities can suppress the defective differentiation and overgrowth phenotype induced by *mei-P26* mutants

Given that *mei-P26* mutants display defective differentiation, we investigated whether the misregulation of Tor activity and RiBi are responsible for the observed phenotypic changes. We modulated Tor and RiBi activities in *mei-P26-*depleted animals, aiming to restore the uncoupled metabolic environment that is characteristic of differentiation: high translation with low RiBi. As genetic manipulation of RiBi results in either a failure in differentiate or premature loss of GSCs,^8^ we took advantage of the small-molecule RNA Pol I inhibitor BMH-21, which has been shown to suppress rRNA synthesis in mammalian cells.^36^ To test the effect of BMH-21 treatment on *Drosophila* ovaries, we used an assay based on the incorporation of 5-ethynyl uridine (EU) to image nascent RNA transcription with and without BMH-21 treatment. In controls, the bulk of the EU incorporation signal is generated by the Pol I-mediated transcription of the ribosomal DNA repeats, accumulating in the nucleolus. Consistent with experiments in mammalian cells, we found that 100 μM BMH-21 treatment was sufficient to dramatically reduce transcription from the nucleolus in *Drosophila* ovaries (Figure S3).

**Figure 5:**
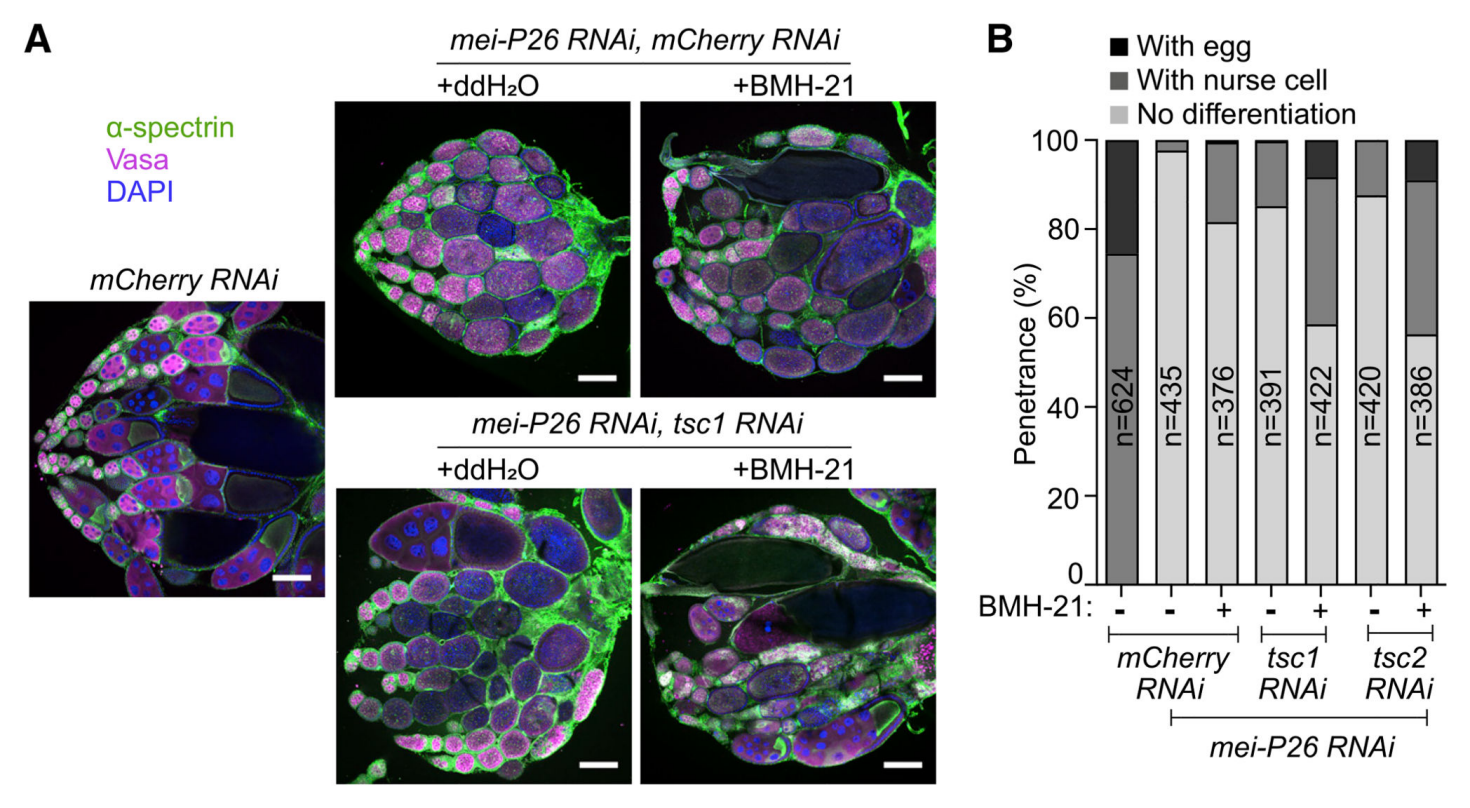
Manipulating RiBi and Tor activity in *mei-P26* ovaries can promote terminal differentiation (A) Ovaries of control (*mCherry RNAi*), *mei-P26 mCherry* double KD (*mei-P26 RNAi, mCherry RNAi*), and *mei-P26 tsc1* double KD (*brat RNAi, tsc1 RNAi*) flies, with or without BMH-21 feeding. Stained with α-spectrin (green), Vasa (magenta), and DAPI (blue). (B) Penetrance of phenotypes of control (*mCherry RNAi*), *mei-P26 mCherry* double KD (*mei-P26 RNAi, mCherry RNAi*), *mei-P26 tsc1* double KD (*mei-P26 RNAi, tsc1 RNAi*), and *mei-P26 tsc2* double KD (*mei-P26 RNAi, tsc2 RNAi*) with or without BMH-21 injection. n is the number of ovarioles analyzed. ***p < 0.0001, chi-square test. Scale bars, 200 μm (A).

We used BMH-21 treatment to test the effect of RiBi suppression on *mei-P26*-depleted germ cells. We observed that abdominal injection of BMH-21 into adult flies led to a moderate suppression of the *mei-P26* KD-induced block in germline differentiation, with ~18% of ovarioles (*mei-P26* RNAi, *mCherry* RNAi + BMH-21) containing terminally differentiated egg chambers (Figures 5A and 5B). A similar suppression effect was observed by genetically activating the Tor kinase during germline development by knocking down Tsc1 or Tsc2 (*mei-P26* RNAi, *tsc1* RNAi, or *tsc2* RNAi), resulting in 12%–15% of ovarioles with terminally differentiated egg chambers (Figures 5A and 5B). When RiBi was suppressed at the same time as activating Tor, robust terminal differentiation was observed in ~40% of ovarioles, and ~10% of ovarioles contained fully developed eggs (Figures 5A and 5B). These experiments show that the block in differentiation phenotype observed in the *meiP26* mutant can be significantly suppressed by redressing levels of Tor activation and RiBi, suggesting that the uncoupled state is essential for proper differentiation.

### Brat is required for Tor activation during NB differentiation

**Figure 6:**
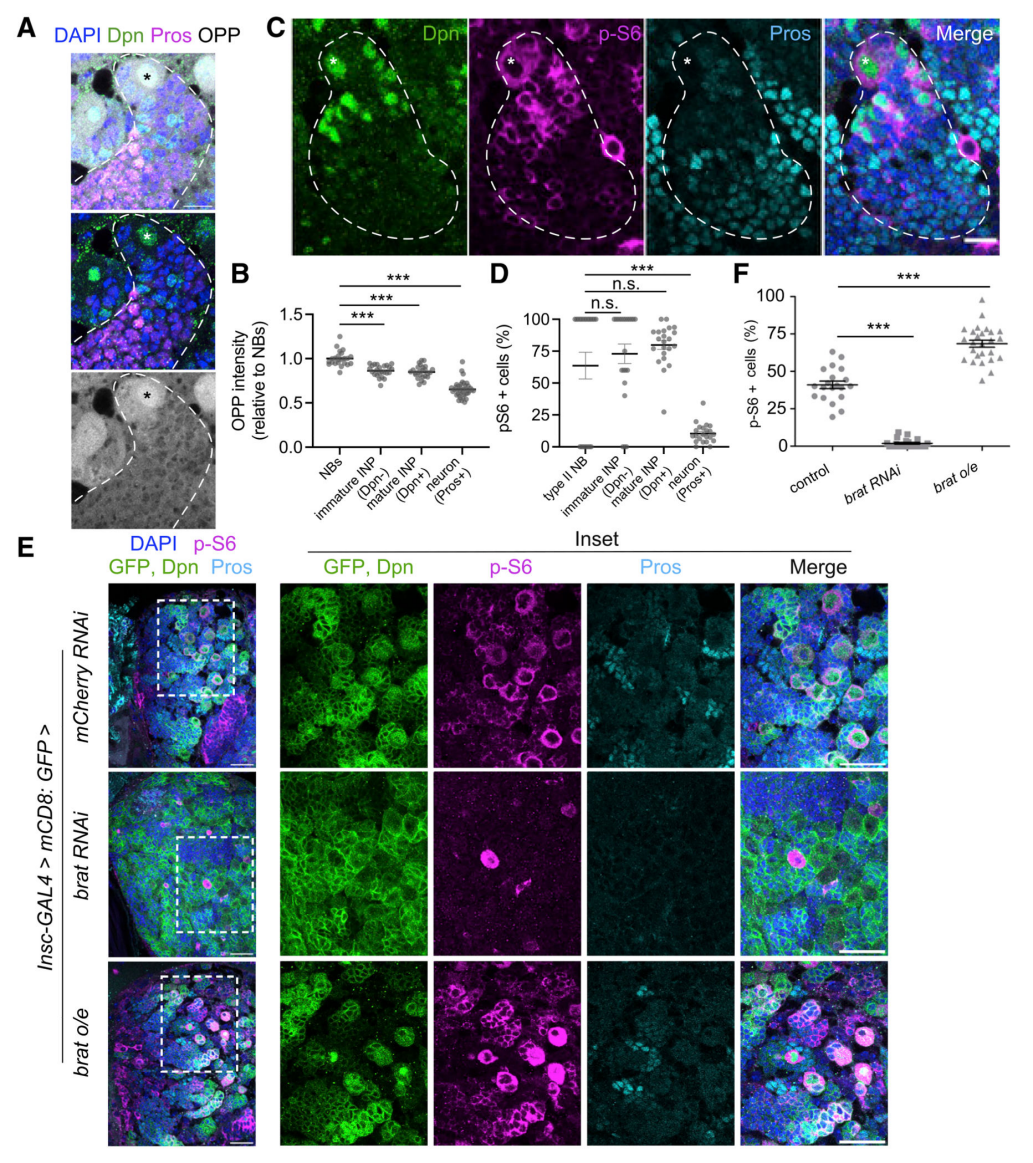
Brat is required for Tor activation during type II NB differentiation (A) A type II NB lineage in the L3 larval brain, stained with DAPI (blue), Dpn (self-renewal marker, green), Pros (pro-differentiation marker, magenta), and OPP (greyscale). Dashed line surrounds a type II NB lineage, asterisk indicates a type II NB (A and C). (B) Quantitation of OPP incorporation in the type II NB lineage, normalized to average NB intensity in each image. Each point represents one cell. (C) A type II NB lineage in the L3 larval brain, stained with DAPI (blue), Dpn (green), p-S6 (Tor activity, magenta), and Pros (cyan). (D) Proportion of p-S6^+^ cells during type II NB differentiation. Each point is a single cluster analyzed, and each cluster contains a single type II NB. (E) Larval brains of control (*mCherry* RNAi), *brat* RNAi, or *brat* overexpression (o/e) driven by *insc-GAL4*, stained with GFP (*insc>mCD8:GFP*, green), Dpn (same channel, green), p-S6 (magenta), Pros (cyan), and DAPI (blue). White dashed rectangles (left) indicate the sources of the insets (right). (F) Percentage of p-S6^+^ GFP^+^ in control (*mCherry RNAi*), *brat* RNAi, and *brat* o/e larval brains. Data are mean ± SEM. ***p < 0.0001, one-way ANOVA followed by Dunnett’s multiple comparisons test (B, D, and F). Scale bars, 10 μm (A and C) or 25 μm (E).

To determine whether TRIM-NHL proteins regulate Tor activity in other stem cell differentiation systems, we examined the type II NBs during larval neurogenesis. In this system, the Mei-P26 paralog, Brain tumor (Brat), also promotes differentiation while downregulating RiBi activity.^5,37^ In the developing larval brain, Brat activity is restricted to differentiating progeny of the type II NBs by protein segregation during an asymmetric cell division.^5,20^ As in the case of GSCs, NBs are characterized by a larger cell size and a higher RiBi rate compared with their differentiating progeny, which include immature intermediate neural progenitors (INPs), mature INPs, ganglion mother cells (GMCs) and neurons (Figure S4A).^5,38^ To determine how translation changes during NB differentiation, we performed the OPP incorporation assay in larval brains. We found that OPP incorporation was highest in the NBs (large cells; Deadpan, Dpn^+^) and INPs (immature INPs: Dpn^−^; Prospero, Pros^−^, mature INPs: Dpn^+^, Pros^−^), and reduced in neurons (Pros^+^) (Figures 6A and 6B). Next, we examined Tor activation during type II NB differentiation using immunofluorescence to detect p-S6. We detected p-S6 in ~64% of type II NBs, ~73% of their immediate progeny cells, and ~80% of mature INPs. However, fewer than 11% of differentiated neurons were positive for p-S6 (Figures 6C and 6D). We examined the effect of rapamycin treatment on p-S6 in the type II lineage. We found that 20 min of rapamycin treatment during *ex vivo* brain culture led to substantial loss of p-S6 signal in the NBs and INPs (Figure S4B). Therefore, we conclude that Tor activity is developmentally regulated during type II NB differentiation.

We tested whether Brat is required for Tor activation during NB differentiation, using *brat* RNAi driven by *insc*-Gal4, which drives in NBs and INPs. Brat functions in the immature INPs to promote INP maturation and prevent reversion to an NB fate,^39^ therefore *brat* KD leads to an accumulation of supernumerary NBs. Although the *brat* KD brain was filled with NB-like cells, we found that the p-S6 signal was mostly abolished in *brat*-deficient brains (Figures 6E and 6F), becoming restricted to a small number of individual cells, which were labeled by mCD8:GFP driven by insc-GAL4. To test whether Brat is sufficient to promote Tor activation, we investigated the expression of p-S6 in larval brains with Brat overexpression. In this context, the p-S6 expression domain was robustly and ectopically expanded into GMCs and newly differentiated neurons (Figures 6E and 6F). These results demonstrate that, analogous to the role of Mei-P26 in GSC differentiation, Brat activates the Tor kinase alongside its known role of downregulating RiBi during differentiation.^5,37^

### The phenotype of *brat* deficiency in larval brains can be suppressed by redressing RiBi and Tor activity

We showed that the phenotype of *mei-P26* mutants in the ovary can be rescued by simultaneously suppressing RiBi and activating Tor, to restore the metabolic uncoupling that is characteristic of differentiation. Therefore, we investigated whether modulating Tor and RiBi activities in the larval brain could also circumvent the block to differentiation observed in *brat-*depleted brains. *brat* depletion leads to the accumulation of undifferentiated Dpn^+^ Pros^−^ cells. Clonal analyses using the *brat*^*11*^ mutation revealed that KD of the Tor-antagonizing regulators Tsc1 or Tsc2 (Figure 2A) was sufficient to restore differentiation (Dpn^−^ Pros^+^ cells) in the absence of Brat in about 10% of clones (2 in 15 clones for *tsc1* RNAi or 3 in 31 clones for *tsc2* RNAi, Figure S5A).

**Figure 7:**
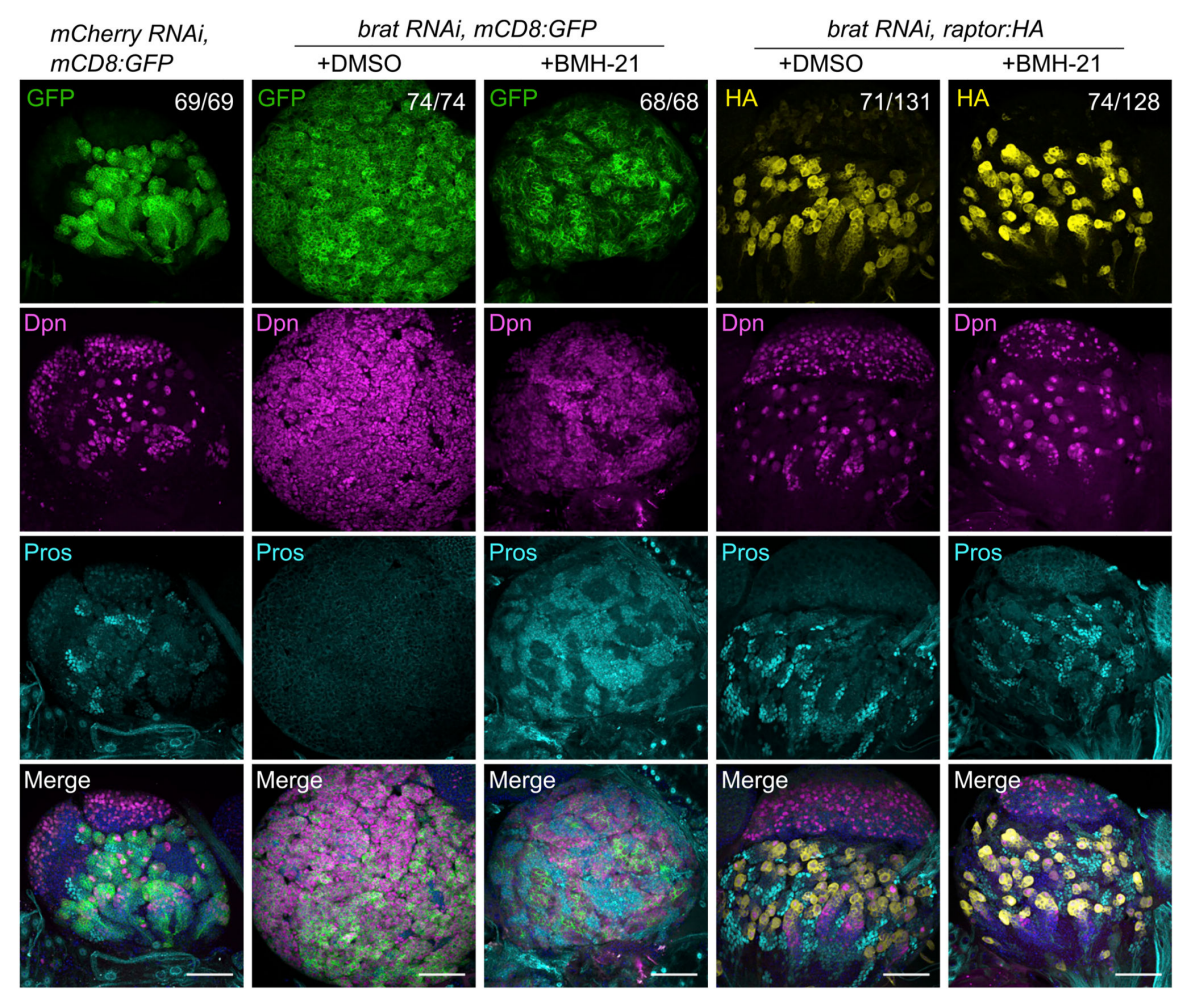
Redressing RiBi and Tor activity promotes terminal differentiation in *brat* larval brains (A) Larval brain lobes of control (*mCherry RNAi, mCD8:*GFP), *brat* KD (*brat RNAi, mCD8:*GFP), and *brat* KD with *raptor* overexpression (*brat RNAi, UAS-raptor:HA*) flies (driven by *insc-GAL4*) with or without BMH-21 feeding. Samples were stained with GFP (green), HA (yellow), Dpn (magenta), Pros (cyan), and DAPI (blue). Numbers in the top panel indicate the penetrance of phenotypes out of three independent experiments. Scale bars, 50 μm.

Moreover, we found that increasing Tor activity through over-expression of Raptor (Figure 2A) in a *brat* RNAi context was sufficient to decrease the undifferentiated Dpn^+^ Pros^−^ population and to promote differentiation (Dpn^−^ Pros^+^ cells; Figure 7). Indeed, in *brat* RNAi larvae with Raptor OE, ~50% of brain lobes had no apparent phenotypic abnormalities (Figures 7, S5B, and S5C). This cellular rescue resulted in a functional recovery: climbing assays revealed that while *brat*-deficient flies had reduced mobility, adult flies overexpressing Raptor in a *brat* RNAi context were indistinguishable from control flies (Figures S6A and S6B; supplemental information; Video S1). These results show that Tor activation can promote the differentiation of *brat*-deficient NBs.

To determine whether inhibition of RiBi has a similar effect on the differentiation of *brat*-deficient NBs, we fed larvae with BMH-21. *brat* RNAi brains showed partial differentiation after BMH-21 feeding, with increased expression of the differentiation marker Pros (Figures 7, S7A, and S7B). However, the distribution of cellular markers did not fully recapitulate the wild-type pattern and we observed numerous cells positive for both Pros and Dpn markers, suggesting that the canonical program of type II NB differentiation was not fully restored (Figure S7B, yellow arrows). To examine the effect of genetic RiBi manipulation, we knocked down Nop60B, a pseudouridine synthase involved in rRNA processing. Remarkably, *Nop60B* KD suppressed the accumulation of Dpn^+^ Pros^−^ NB-like cells observed in *brat* KD and restored the differentiation pathway to produce Dpn^−^ Pros^+^ cells (Figure S7C).

Collectively, our results demonstrate that TRIM-NHL proteins drive terminal differentiation by simultaneously suppressing RiBi and promoting translation, uncoupling two of the most energy-consuming biosynthetic activities.

## Discussion

Accumulating evidence indicates that dynamic regulation of cellular metabolism plays a key role at the tissue level in development and homeostasis.^1,3^ During stem cell differentiation, RiBi and protein synthesis rates are actively regulated, and these dynamic changes are essential for the balance of self-renewal, growth, and differentiation.^4^ Here, we have shown that two members of the TRIM-NHL protein family (Mei-P26 and Brat) are responsible for the simultaneous regulation of RiBi and Tor kinase activity/translation during stem cell differentiation, in GSC and NB systems. The antagonistic regulation of these highly energy-consuming processes creates a state of high translation with low RiBi, which deviates from the canonical growth paradigm downstream of Tor activation.^17^ We find that the differentiation block in *mei-P26/brat* mutants can be rescued by modulating translation and RiBi to restore the uncoupled state, suggesting that metabolic uncoupling is a driver of terminal differentiation.

We have shown that Mei-P26 and Brat uncouple translation and RiBi specifically during differentiation. However, both Mei-P26 and Brat are expressed in the stem cells as well as their differentiating progeny,^7,20^ so it remains an open question how their activity is restricted to the differentiating cells. Brat is transcribed and translated in NBs, but is sequestered to the basal membrane and segregated into differentiating daughter cells.^20^ This mechanism enriches Brat in the INP compared with the NB and perhaps this higher concentration is necessary for Brat action. In contrast, Mei-P26 is equally expressed between GSCs and differentiating cells,^7^ suggesting that a different regulatory mechanism is at play in the GSCs to limit Mei-P26 activity. Interestingly, we have shown that overexpression of Mei-P26 is sufficient to drive ectopic Tor activation in the GSCs, suggesting that the as yet unknown factor that suppresses Mei-P26 activity in GSCs becomes overwhelmed in a Mei-P26 overexpression condition.

In any case, the presence of Mei-P26 protein from the GSC stage may allow for the prompt modulation of its downstream targets in the first stages of differentiation. Our results indicate that Mei-P26 acts very rapidly in the early stages of differentiation, regulating two different PIKK members in opposite directions: promoting translation through activating Tor, and suppressing RiBi through TRRAP. Mei-P26 upregulates the activity of the Tor kinase but the molecular mechanisms of its interaction with TRRAP are unknown. Interestingly Tor is activated in CBs, but nucleolar volume reduction is only apparent from the 2-cell cyst stage onward,^8^ which may be explained by the rapid action of the Tor kinase compared with TRRAP, which is a transcriptional regulator.^35^

We found that both the TRIM motif and NHL repeats of Mei-P26 are required for the regulation of the Tor kinase. The NHL repeats of Brat and Mei-P26 form a β propellor structure^30,31,33^ (Figure 4B), and a similar structure is also found in all components of the GATOR2 complex (Nup44A, Sec13, Mio, Wdr24, and Wdr59), which activates the Tor kinase during GSC differentiation. Moreover, like Mei-P26, Wdr24 and Wdr59 also contain RING domains. The recently published structure of the human GATOR2 revealed that the complex is held together through interactions of these RING domains, while the β propellors interact with the amino acid sensor Sestrin and the downstream GATOR1 complex.^40^ In line with our finding that the Mei-P26 E3 ubiquitin ligase activity is not required for TOR activation, the E3 ubiquitin ligase activity of the human GATOR2 RING domains was shown to be dispensable for activation of Tor. Although awaiting further biochemical verification, we speculate that the Mei-P26 RING domain and β propellor allow interaction with the GATOR2 complex to modulate the activity of the Tor pathway. Notably, Brat lacks the RING domain of the TRIM motif^30,41^ and yet activates Tor and suppresses RiBi in the brain, which could imply that Brat and Mei-P26 act through different molecular interactions. Indeed, Brat is expressed in the ovary, alongside Mei-P26 in differentiating GSC progeny, where it has a role in cell size and differentiation,^42^ but is unable to compensate when Mei-P26 is knocked down. It is unclear how Mei-P26 and Brat may interact in the ovary and it will be important to build a more complete molecular picture of Mei-P26 and Brat activity.

Unlike in GSCs, Tor is active in NBs. Upon *brat* KD, INPs revert to an NB-like fate,^39^ but we observed that Tor activity was not maintained, except in a small number of cells. Brat is asymmetrically segregated into the differentiating daughter cells, so we would expect that Tor activity in the type II NBs would be Brat independent. However, most of the NB-like cells in the *brat* KD do not express p-S6, unlike the wild-type NBs. This might indicate that either the Tor activity in wild-type NBs is dependent on Brat, or that the supernumerary NBs originating from reversion of INPs in the *brat* KD are lacking a Brat-independent intrinsic or extrinsic signal that activates Tor in the wild type. In late-stage larvae, Tor activation in the type I NB is independent of insulin or amino acid signaling, and is instead activated by ligands expressed from the glial niche.^43^ In this so-called brain-sparing mechanism, glial signaling secures NB growth and division regardless of nutritional status, ensuring the successful production of the complete brain during larval development. Perhaps the supernumerary NBs in the *brat* KD do not establish a glial niche to receive these signals. In sharp contrast to the brain-sparing process, GSC growth and proliferation is acutely dependent on the nutritional state of the animal to maximize survival of the offspring.^44^ Therefore, it is not surprising that we find a role for amino acid sensing in the activation of Tor kinase during GSC differentiation.

Phosphorylation of the ribosomal p-S6 is used here as a readout of Tor kinase activity, but the Tor kinase has several other known downstream targets with different functions. 4E-BP is also phosphorylated by Tor kinase and p4E-BP has been shown previously to be low during early cyst differentiation (although with a small frequency of positive cells, consistent with changes during the cell cycle), with levels rising later, in post-meiotic cysts.^45^ Taken together with our data, this suggests that the Tor kinase differentially regulates its targets during germline development. Given that the *Tor* KD phenotype causes cyst death at the 8- or 16-cell cyst stages,^21^ while the *S6K* KD shown in Figure 2 does not exhibit significant phenotypic defects in differentiation, there must be additional effectors of the Tor kinase that are coregulated with p-S6.

Tor kinase activity is generally associated with growth,^17^ but interestingly in both GSC and NB systems differentiation is accompanied by a decrease in cellular volume while Tor is active.^7,46^ Furthermore, *mei-P26* and *brat* mutants display tumor phenotypes of large, undifferentiated cells despite the loss of Tor activity.^5,7^ Canonically, Tor activation is associated with an increase in both translation and RiBi, creating an optimal scenario to drive growth.^17^ However, in the stem cell systems described here, Tor activity is uncoupled from RiBi such that limited ribosomes are produced even while Tor is active. This lack of ribosome generation may act as a brake on Tor-driven growth. We must also consider that the observed changes in cell size are affected by both cell growth and division. Tor has been shown to promote germline cyst proliferation.^21^ In the *mei-P26*/*brat* tumor scenario, the loss of Tor may slow cell divisions such that cells have more time to grow, while the increased RiBi rate might allow growth to continue beyond its usual depletion of resources. This combination would result in larger cells despite a lower growth rate.

The relationship between growth and cell cycle may also provide insight into how uncoupling of translation and RiBi drives differentiation. Cell cycle exit is a prerequisite for terminal differentiation in both the GSC and NB systems.^38,47^ In general, the coordination of anabolic activities is required for sustained growth and proliferation,^48^ so perhaps uncoupling translation and RiBi limits the possible growth and number of cell divisions prior to exhaustion of cellular resources. In the pupal brain, type I NB growth is limited such that cells shrink with each division and this shrinkage induces the final symmetric division that leads to terminal differentiation of both daughters.^49,50^ In the GSC and larval NB systems studied here, anabolic uncoupling may start a timer before resource depletion leads to terminal differentiation. It is possible that depletion of a general resource, such as functioning ribosomes, directly leads to differentiation as a default state when the cells become unable to maintain divisions. Careful analysis of proliferation rates and cell size, perhaps using long-term live imaging, will be needed to unpick the contributions of growth and division. Alternatively, another possibility is that the depletion of general resources results in a specific signal (e.g., a protein or free ribosomal subunits) reaching a critical concentration to drive differentiation.

Altogether, our work uncovers a hitherto overlooked role of metabolic imbalance in driving differentiation. As RiBi and translation rates are known to be dynamically regulated in many different stem cell systems, we propose that this mechanism has general implications for our understanding of differentiation.

### Limitations of the study

In this study, we have used phosphorylation of RpS6 as a down-stream readout of Tor activity and therefore we may not capture activity of Tor that does not go through the S6 kinase. In examining the genetic pathway upstream of Tor, we have used knockdowns rather than mutants, which may lead to an incomplete removal of protein. Finally, the rescue experiments using *brat* mutant clones in the brain with *tsc1* or *tsc2* KD were only partially penetrant, suggesting additional factors required for robust NB differentiation in the *brat* mutant background may be missing.

## Star ⋆ Methods

### Key Resources Table

**Table:** 

REAGENT or RESOURCE	SOURCE	IDENTIFIER
Antibodies		
rat anti-Dpn (1:200)	Abcam	(Abcam Cat# ab195173, RRID: AB_2687586)
Mouse anti-Fib (1:200)	Abcam	(Abcam Cat# ab4566, RRID: AB_304523)
Rabbit anti-HA (1:100)	Abcam	(Abcam Cat# ab9110, RRID: AB_307019)
Rat anti-HA (1:100)	Sigma aldrich	(Roche Cat# 3F10, RRID: AB_2314622)
Rat anti-GFP (1:200)	Millipore	(Millipore Cat# MAB3580, RRID: AB_94936)
Chicken anti-GFP (1:1000)	Aves Labs	(Aves Labs Cat# GFP-1010, RRID: AB_2307313)
Rat anti-RFP (1:200)	Chromotek	(ChromoTek Cat# 5f8-100, RRID: AB_2336064)
Mouse anti-a-spectrin (1:100)	DSHB	(DSHB Cat# 3A9 (323 or M10-2), RRID: AB_528473)
Mouse anti-Pros (1:20)	DSHB	(DSHB Cat# Prospero [MR1A], RRID: AB_528440)
Rabbit ant-phosphorylated-S6 (1:200)	Aurelio Teleman (Romero-Pozuelo et al., 2017)^23^ and this study	N/A
Rabbit anti-Vasa (1:5000)	Ruth Lehmann	N/A
Rabbit anti-Mei-P26 (1:1000)	Paul Lasko	N/A
Goat anti-mouse Alexa 488 (1:200)	Invitrogen	(Thermo Fisher Scientific Cat# A32723, RRID: AB_2633275)
Donkey anti-rat Alexa 488 (1:200)	Invitrogen	(Thermo Fisher Scientific Cat# A-11006, RRID: AB_2534074)
Goat anti-rabbit Alex 568 (1:200)	Invitrogen	(Thermo Fisher Scientific Cat# A-11011, RRID: AB_143157)
Donkey anti-mouse Cy3 (1:200)	Jackson ImmunoResearch	(Jackson ImmunoResearch Labs Cat# 715-165-150, RRID: AB_2340813)
Donkey anti-rabbit Alex647 (1:200)	Jackson ImmunoResearch	(Jackson ImmunoResearch Labs Cat# 711-605-152, RRID: AB_2492288)
Goat anti-mouse Alexa 647 (1:200)	Invitrogen	(Thermo Fisher Scientific Cat# A-21236, RRID: AB_2535805)
Chemicals, peptides, and recombinant proteins		
BHM-21	Sigma Aldrich	SML1183
rapamycin	Sigma Aldrich	R0395
TRIzol Reagent	Invitrogen	15596026
Formaldehyde	Thermo Fisher Scientific	28908
VectaShield medium	Vector Laboratories	H1000
Critical commercial assays		
Click-iT Plus OPP Alexa Fluor 594 Protein Synthesis Assay Kit	Invitrogen	C10457
Click-iT RNA Alexa Fluor 594 Imaging Kit	Invitrogen	C10330
Qubit RNA HS Assay Kit	Invitrogen	Q32852
NEBNext Poly(A) mRNA Magnetic Isolation Module	New England Biolabs	E7490S
NEBNext Multiplex Oligos for Illumina	New England Biolabs	E7335S, E7500S
NEBNext Ultra Directional RNA Library Prep Kit for Illumina	New England Biolabs	E7490S
Deposited data		
RNA-seq for Mei-P26 OE and RNAi in *bam*^*Δ86*^ background	This study	GEO: GSE218205
Experimental models: Organisms/strains		
*Drosophila: w* ^*1118*^	Lehmann lab stock	N/A
*Drosophila: UAS-Dcr2, w* ^*1118*^ *; nosP-GAL4-NGT40*	Bloomington Drosophila Stock Centre (BDSC)	RRID: BDSC_25751
*Drosophila: P{bamP-GFP}*	Chen and McKearin, 2003^51^	N/A
*Drosophila:;; bam* ^*Δ86*^ *, ry, e/TM3, Sb, ry, e*	McKearin and Ohlstein, 1995^52^	N/A
*Drosophila: y*^*1*^ *w*^*1*^ *mei-P26*^*mfs1*^*;;; Dp(1;4)A17/sv*^*spa-pol*^	BDSC, Page et al., 2000^19^	RRID: BDSC_25919
*Drosophila: w* ^*1118*^ *; UASp-mei-p26.N*	BDSC	RRID: BDSC_25771
*Drosophila: brat* ^*11*^ *; insc-Gal4*	BDSC	RRID: BDSC_8751
*Drosophila: UAS-mCD8:GFP*	BDSC	RRID: BDSC_5130 and RRID: BDSC_5137
*Drosophila:* UASp-*brat*	Harris et al., 2011^42^	N/A
*Drosophila: UAS-raptor-HA*	BDSC	RRID: BDSC_53726
Drosophila: w*; FRT 40A	BDSC	RRID: BDSC_86317
*Drosophila: hs-FLP, UAS-mCD8: GFP; tubP-GAL80, FRT 40A; tubP-GAL4*	BDSC	RRID: BDSC_44406 and RRID: BDSC_84300
*Drosophila: w;; [FlyFos020668(Tor29074::2XTY1-SGFP-V5-preTEV-BLRP- 3XFLAG)dFRT] VK00033*	VDRC	#318201
*Drosophila: w;; [FlyFos022619(raptor[16724]::2XTY1-SGFP-V5-preTEV-BLRP-3XFLAG)dFRT]VK00033*	VDRC	#318149
*Drosophila:*;;PBac{fTRG00888.sfGFP-TVPTBF}VK00033 (Nop60B::GFP)	VDRC	#318245
*Drosophila: y*^*1*^ sc^*^v^*1*^ *sev*^*21*^*; P{VALIUM20- mCherry}attP2* (mCherry RNAi)	BDSC	RRID: BDSC_35785
*Drosophila: y*^*1*^ sc* *v*^*1*^*; P{TRiP.GL01124}attP40* (mei-P26 RNAi)	BDSC	RRID: BDSC_36855
Reagent of resource cell: Drosophila: y1 sc* v1 sev21; P{y+t7.7 v+t1.8= TRiP.HMS01121}attP2 (brat RNAi)	BDSC	RRID:BDSC_34646
*Drosophila: y*^*1*^ sc^*^ *v*^*1*^*; P{TRiP.GL00012}attP2* (tsc1 RNAi)	BDSC	RRID: BDSC_35144
*Drosophila: y*^*1*^ sc^*^ *v*^*1*^*; P{TRiP.GL00321}attP2* (tsc2 RNAi)	BDSC	RRID: BDSC_35401
Drosophila: y[1] sc[*] v[1] sev[21]; P{y[+t7.7] v[+t1.8] = TRiP.GL00139}attP2 (InR RNAi)	BDSC	RRID: BDSC_35251
*Drosophila:* y^1^ sc* v^1^ sev^21^; P{TRiP.GL00525}attP40 (chico RNAi)	BDSC	RRID: BDSC_36788
*Drosophila: y[1] v[1]; P{y[+t7.7] v[+t1.8] = TRiP.HMS00007}attP2* (Akt RNAi)	BDSC	RRID: BDSC_33615
Drosophila: y[1] sc[*] v[1] sev[21]; P{y[+t7.7] v[+t1.8] = TRiP.HMS01064}attP2 (RagAB RNAi)	BDSC	RRID: BDSC_34590
Drosophila: y[1] sc[*] v[1] sev[21]; P{y[+t7.7] v[+t1.8] = TRiP.GL01327}attP2 (s6k RNAi)	BDSC	RRID: BDSC_41895
Drosophila: y[1] sc[*] v[1];; P{TRiP.HMS00904}attP2 [Tor] (Tor RNAi)	BDSC	RRID: BDSC_33951
Drosophila: y[1] sc[*] v[1] sev[21]; P{y[+t7.7] v[+t1.8] = TRiP.HMC05152}attP40 (Pi3K92E/Dp110 RNAi)	BDSC	RRID: BDSC_61182
Drosophila: y[1] sc[*] v[1] sev[21]; P{y[+t7.7] v[+t1.8] = TRiP .HMS00261}attP2/TM3, Sb[1] (Pi3K59F/Vps34 RNAi)	BDSC	RRID: BDSC_33384
Drosophila: y[1] sc[*] v[1] sev[21]; P{y[+t7.7] v[+t1.8] = TRiP.GL00156}attP2 (LexA RNAi)	BDSC	RRID: BDSC_67945
Drosophila: y[1] sc[*] v[1] sev[21]; P{y[+t7.7] v[+t1.8] = TRiP.HMC04815}attP40 (nop60B RNAi)	BDSC	RRID: BDSC_57500
*Drosophila:* y[1] v[1]; P{TRiP.HMS01825}attP40 (Nup44A RNAi)	BDSC	RRID: BDSC_38357
Drosophila: y[1] w*; M{3xP3-RFP.attP’}ZH-51C[UASp-Mei-P26.wt, mini-w+] (UASp-Mei-P26)	This study	N/A
Drosophila: y[1] w*; M{3xP3-RFP.attP’}ZH-51C[UASp-Mei-P26.dNHL, mini-w+] (UASp-Mei- P26_ΔNHL)	This study	N/A
Drosophila: y[1] w*; M{3xP3-RFP.attP’}ZH-51C[UASp-Mei-P26.dTRIM, mini-w+] (UASp-Mei-P26_ΔTRIM)	This study	N/A
Drosophila: y[1] w*; M{3xP3-RFP.attP’}ZH- 51C[UASp-Mei-P26.P224A, mini-w+] (UASp-Mei-P26_*E3ligaseDEAD*)	This study	N/A
Recombinant DNA		
pWallium22 plasmid	Perkins et al., 2015^53^	N/A
Software and algorithms		
Prism	GraphPad by Dotmatics	N/A
FIJI	Schindelin et al., 2012^54^	N/A
Bowtie2	Langmead & Salzberg, 2012^55^	N/A
STAR	Dobin et al., 2013^56^	N/A
Cufflinks	Trapnell et al., 2010^57^	N/A

### Resource Availability

#### Lead contact

Further information and requests for resources or reagents should be directed to the lead contact Felipe Karam Teixeira (fk319@cam. ac.uk).

#### Materials availability

Transgenic *Drosophila* lines generated in this study are available upon request.

### Experimental Model and Subject Details

#### Drosophila melanogaster

Unless stated otherwise, stocks and crosses were maintained on standard propionic food at 25°C. For rapamycin feeding experiments with adult flies, 200 μL 100 μM rapamycin (Sigma Aldrich) was added to the top of food at least one day before newly eclosed flies were transferred into the vial. Flies were raised in food containing rapamycin for 3 days at 25°C before ovary dissections. For feeding experiments with larvae, 50 μL 2 mM BMH-21 (Sigma Aldrich) was added to the top of food for no less than one day before the experiment. Three-day-old larvae grown at room temperature were transferred to the food containing BMH-21 and raised at 29°C for three days before brain dissections. For BMH-21 injection in adult flies, 69 nL 200 μM BMH-21 diluted in double-distilled water was injected into the abdomen of one-day-old females. Injected flies were raised for five days at 25°C before ovary dissections.

*Drosophila melanogaster* stocks and transgenes used are listed in the key resources table. The following stocks were generated for this study: Mei-P26 full length and deletion constructs, with a UASp promoter (pWalium22 backbone;^53^) inserted with PhiC31 integration at the attP51C site on chromosome 2R. All constructs included an N-terminal HA-tag. The constructs (depicted in Figure 4A) were full length Mei-P26, ΔNHL (a deletion of the C-terminal 344 amino acids), ΔTRIM (a deletion of the N-terminal 615 amino acids) and E3ligase DEAD (substituting proline 224 with alanine).

### Method Details

#### Immunofluorescence and antibodies

The antibodies used are listed in the key resources table. Adult ovaries were dissected in cold PBS buffer and fixed in PBST (PBS with 0.2% Triton X-100) containing 4% Formaldehyde (Thermo Fisher Scientific) for 30 min. Larval brains were dissected in Schneider’s insect medium and transferred immediately into cold fixative (4% formaldehyde in PBST), then fixed for 25 min. Fixed tissues were rinsed three times with PBST before incubation in blocking buffer (PBS with 5% goat serum) overnight at 4°C. Samples were then incubated with primary antibody diluted in blocking buffer overnight at 4°C, washed four times with blocking buffer, and incubated with secondary antibodies and DAPI diluted in blocking buffer overnight at 4°C. Samples were washed four times with PBST and mounted in VectaShield medium (Vector Laboratories). Fluorescent images were acquired on a Leica SP8 confocal microscope using a 40X oil objective or a 20× dry objective. Images were processed using Fiji.^54^

#### *ex vivo* brain culture

L3 larval brains were dissected in culture media (80% Schneider’s Insect Medium, 20% Fetal Bovine Serum and larval extract (according to (Hailstone et al., 2020) but with the omission of insulin). For rapamycin treatment, after dissection, brains were incubated at for 20 min room temperature in culture media with or without 10 μM rapamycin. For OPP incorporation assay, dissected brains were incubated with OP-puro 50 μM for 30 min. In either case, brains were then fixed and stained according to standard procedures described below.

#### Measurement of global protein synthesis *in vivo*

Protein synthesis was detected by the Click-iT Plus OPP Alexa Fluor 594 Protein Synthesis Assay Kit (Invitrogen) as previously described.^8^ Unless stated otherwise, ovaries were dissected in Shields and Sang M3 Insect Medium and were immediately transferred after dissection to fresh medium containing a 1:400 dilution of Click-iT OPP Reagent (OP-puro 50 μM). For the experiment in Figure 1C, dissected ovaries were incubated in media containing 10 μM rapamycin for 30 min before exposure to the Click-iT OPP reagent. Samples were incubated with OPP at room temperature for 30 min, rinsed 3 times with PBS, and fixed with 4% form-aldehyde in PBS for 30 min. After the Click-iT reaction, samples were washed with PBS with 1% BSA and 0.2% Triton X-100 for 1 h and immunostained according to standard procedures. Quantification of OP-Puro fluorescence intensity was performed as previously described^8^ using Fiji. Each experiment was performed at least three times.

#### Measurement of nascent RNA synthesis with BMH-21 treatment

Ovaries were dissected in Shields and Sang M3 Insect Medium. Ovaries were treated for 1 h at RT with 100 μM BMH-21 or DMSO control, in Shields and Sang M3 Insect Medium. We estimate 100 μM BMH-21 to be approximately equivalent to the concentration in the abdomen after injection in our experiments. Media was exchanged to include 10 mM 5-ethynyl uridine (EU) and ovaries were incubated at room temperature for a further 2 h. Ovaries were fixed with 4% formaldehyde in PBS containing 0.3% Triton X-, for 25 min at RT, and then washed 3 times for 15 min each in PBS with 0.3% Triton X-. Nascent RNA was visualized using Click-iT RNA Alexa Fluor 594 Imaging Kit (Invitrogen) according to manufacturer’s instructions.

#### RNA sequencing

120 pairs of ovaries were dissected for each sample and immediately stored at −80°C after dissection. Frozen samples were homogenized in TRIzol Reagent (Invitrogen) using an electrical pestle and further disrupted by passing 15 times through a 26-gauge syringe. Total RNA was isolated using TRIzol Reagent following the manufacturer’s protocol. After RNA quantification using Qubit RNA High Sensitivity Assay Kit (Invitrogen), Poly(A)-selected RNA-sequencing (RNA-seq) libraries were generated using 2.5 μg of purified RNA with the NEBNext Poly(A) mRNA Magnetic Isolation Module and the NEBNext Ultra Directional RNA Library Prep Kit for Illumina. Libraries were multiplexed using the NEBNext Multiplex Oligos for Illumina and sequenced in single-end, 50-nt-long reads on an Illumina HiSeq 2500. The resulting RNA-sequencing data was first aligned to ribosomal RNA using Bowtie2.^55^ Non-rRNA reads were mapped to the *Drosophila melanogaster* genome (dm6) using STAR,^56^ and transcript abundance was quantified and differentially expressed genes were identified using Cufflinks.^57^ Analyses were performed with two samples, each with two biological replicates.

#### Climbing assay

20 1-2 day-old flies were transferred into a fresh vial. The proportion of flies reaching the top of the vial within 20 s after knocking down was recorded. The results represent data collected from six replicates.

#### Nucleolar volume measurement

The volume of Fibrillarin-stained nucleoli was determined based on z stack confocal images using the ‘3D object counter’ Fiji plug-in. Objects on the edge of images were excluded, and threshold and size filters were automatically set. Data were obtained from three independent ovaries.

### Quantification and Statistical Analysis

For RNA-seq analysis, transcript abundance was quantified and differentially expressed genes were identified using Cufflinks,^57^ as above. For other analyses, all experiments were conducted not less than 3 times independently. Statistical analysis was performed using GraphPad Prism software and details are found in the relevant figure legends. Statistical significance (p value) was tested by applying paired *t*-tests with a 95% confidence interval, one-way ANOVA with Dunnett’s multiple comparisons test, or Chi-square test. All error bars represent the standard error of the mean (SEM). No statistical methods were used to predetermine sample size. Experiments were neither intentionally randomized nor intentionally ordered. Investigators were not blinded to allocation during experiments and outcome assessment.

## Supplementary Material

Figures S1-S7

Table S1

Video S1

## Data Availability

RNA-seq data generated during this current study has been deposited at GEO and is publicly available as of the date of publication. Accession number is listed in the key resources table. This paper does not report original code. Any additional information required to reanalyze the data reported in this paper is available from the lead contact upon request.

## In brief

Translation and ribosome biogenesis can be uncoupled during stem cell differentiation. Gui et al. show that Mei-P26 and Brat activate the Tor kinase while repressing ribosome biogenesis in germline and neural stem cell differentiation, respectively. Manipulating Tor activity and ribosome biogenesis restores terminal differentiation in Mei-P26- and Brat-deficient cells.

## Highlights

- Tor is activated during germline stem cell differentiation via the amino acid pathway
- Activating Tor while repressing RiBi creates the conditions for differentiation
- Mei-P26 and Brat activate Tor in the germline and brain, respectively
- Redressing Tor activity and ribosome biogenesis can restore terminal differentiation
