# Supplementary material for: Simultaneous activation of Tor and suppression of ribosome biogenesis by TRIM-NHL proteins promotes terminal differentiation
Source: Cell Rep. Author manuscript; Available in PMC 2025 Feb 25. (PMC7617432; doi:10.1016/j.celrep.2023.112181)
Supplement: Figures S1-S7 [file EMS203439-supplement-Figures_S1_S7.pdf]

Cell Reports, Volume *42*

Supplemental information

Simultaneous activation of Tor and suppression of ribosome biogenesis by TRIM-NHL proteins promotes terminal differentiation

Jinghua Gui, Tamsin J. Samuels, Katarina Z.A. Grobicki, and Felipe Karam Teixeira

Supplementary Data

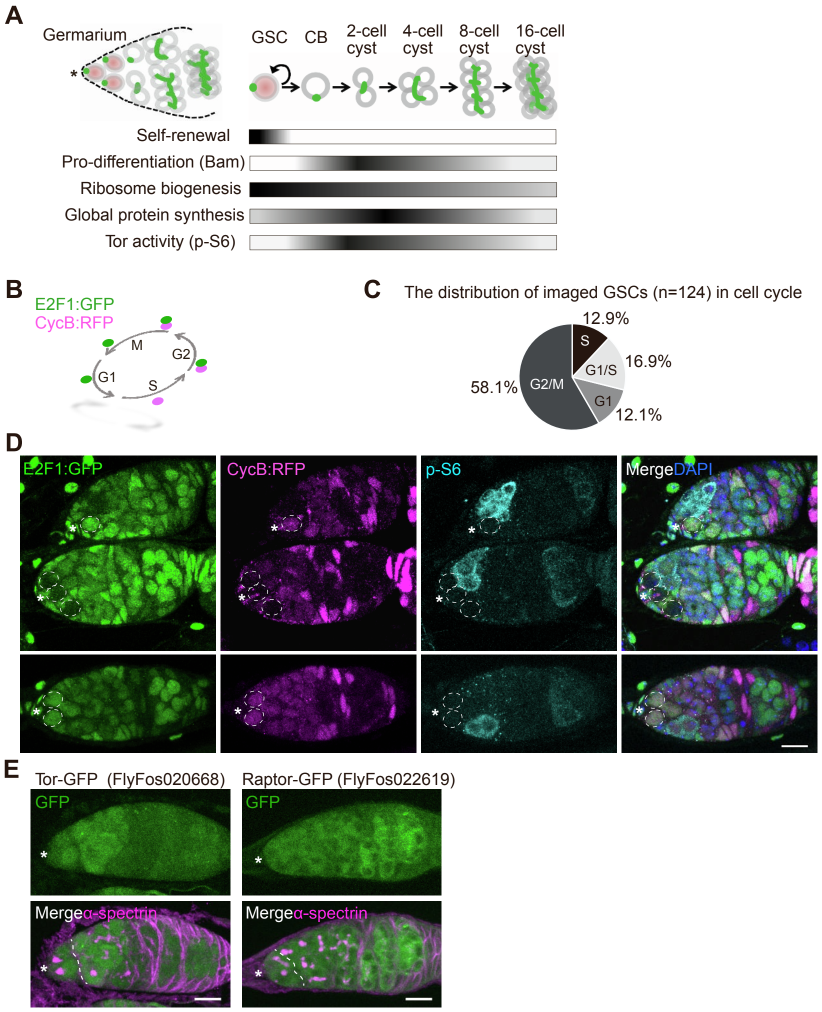

Supplementary figure 1. Tor activation during germline stem cell differentiation. (Related to Figure 1) (A) Schematic of the structure of germarium and stages of GSC differentiation. Spectrosomes/fusomes in green. The asterisk indicates GSC niche. GSCs are marked by pink nuclei. **(B)** Schematic showing the rationale of the two-color fly FUCCI system. **(C)** Representative FUCCI germaria labeled with GFP (E2F1: GFP, green), RFP (CycB:RFP, magenta), p-S6 (Tor activity, cyan), and DAPI (nuclei, blue). Dashed circles mark GSCs. **(D)** Distribution of cell-cycle phase of imaged GSCs (n=124). **(E)** Representative image of germaria of transgenic Tor-GFP (left, FlyFos020668) and Raptor-GFP (right, FlyFos022619) flies labeled with α-spectrin (spectrosomes/fusomes, magenta) and GFP

(fusion proteins, green). Dashed lines indicate the boundary between GSCs and differentiating cells. The asterisk indicates GSC niche (C, E). Scale bars, 10 µm (C, E).

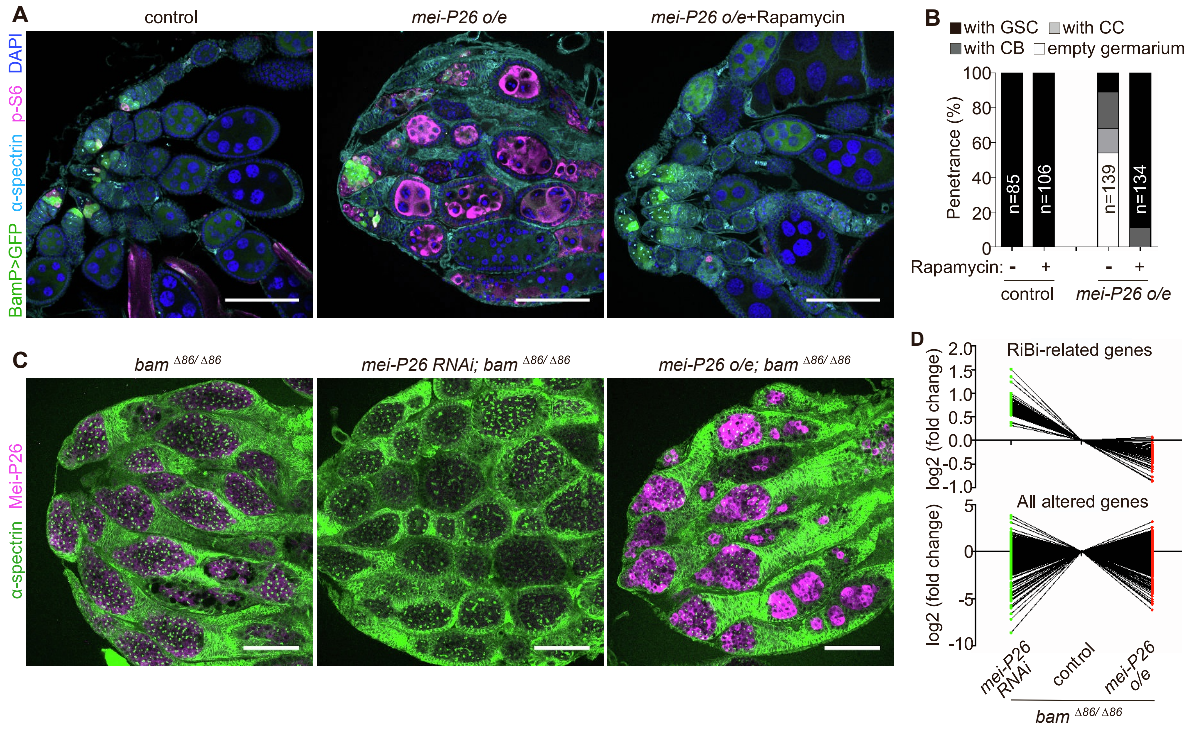

Supplementary figure 2. Mei-P26 activates Tor and represses RiBi-related genes. (Related to Figure 3 and Supplementary Table 1) (A) Representative ovary images of control (*nos-gal4/+*) and Mei-P26 overexpression (o/e) (*nos-gal4/UASp-mei-P26)* flies with or without rapamycin feeding. Ovaries were stained with GFP (BamP>GFP, differentiating cells, green), p-S6 (Tor activity, magenta), α-spectrin (spectrosome/fusome, cyan), and DAPI (nuclei, blue). **(B)** Distribution of germarium phenotypes of control (*nos-gal4/+*) and Mei-P26 o/e (*nos-gal4/UASp-mei-P26)* with or without rapamycin feeding. **(C)** Representative ovary images of control (*nos-gal4/+*), *mei-P26* KD (*nos-gal4/UAS-mei-P26 RNAi)*, and *mei-P26* o/e (*nos-gal4/UASp-mei-P26)* in *bam*$^{Δ86/Δ86}$ background. Samples were stained with α-spectrin (green) and Mei-P26 (magenta). **(D)** RNA profiling of ovaries of control (*nos-gal4/+*), *mei-P26* KD (RNAi, *nos-gal4/UAS-mei-P26 RNAi)*, and *mei-P26* o/e (*nos-gal4/UASp-mei-P26)* in *bam*$^{Δ86/Δ86}$ background. Scale bars, 200 µm (A) or 100 µm (C).

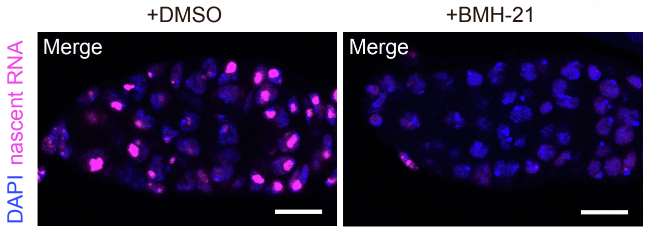

Supplementary figure 3. BMH-21 acts to inhibit rRNA synthesis in the germarium. (Related to Figure 5) Representative confocal images of germaria treated with DMSO or BMH-21. Dissected ovaries were treated with DMSO or 100 μM BMH-21 (we estimate to be approximately equivalent to the concentration in the abdomen after injection in our experiments) for 3 hours, including 5-ethynyl uridine (EU) for the final 2 hours allowing visualization of nascent RNA. In the DMSO control most nascent RNA is generated in the nucleolus, and this is almost entirely abolished with BMH-21 treatment. Image is a max projection of 3 z planes. Scale bars, 10 μm.

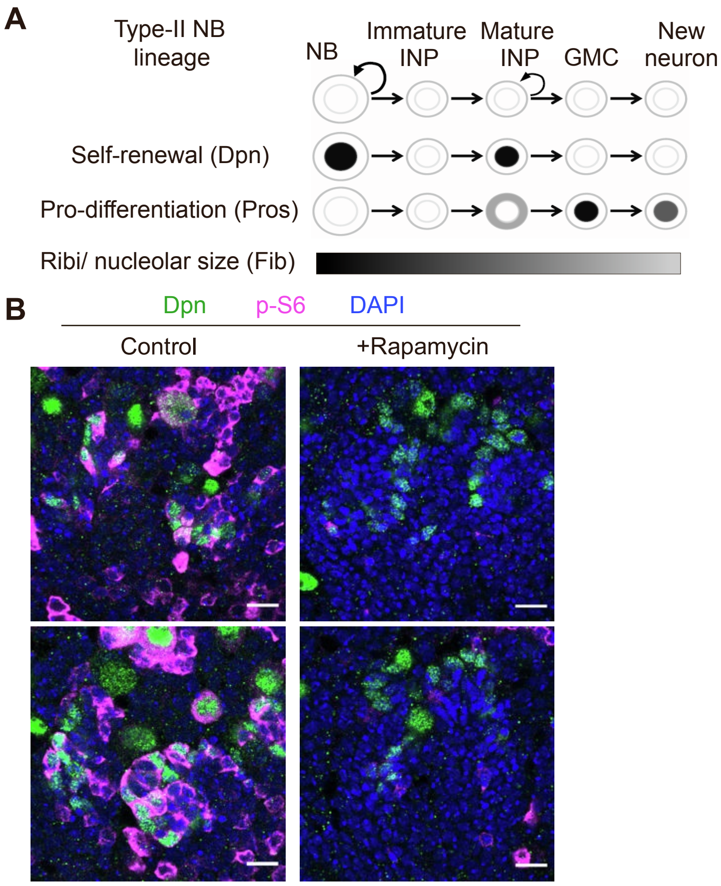

Supplementary figure 4. Rapamycin treatment reduces p-S6 expression in the larval brain. (Related to Figure 6) (A) Schematic showing different cell types and expression markers during type II NB differentiation. **(B)** Representative images of type II NB clusters after 20 minutes of *ex vivo* culture with or without 10 μM rapamycin. Samples are stained with Dpn (NBs and mature INPs, green), p-S6 (magenta) and DAPI (blue). Scale bars, 10 μm.

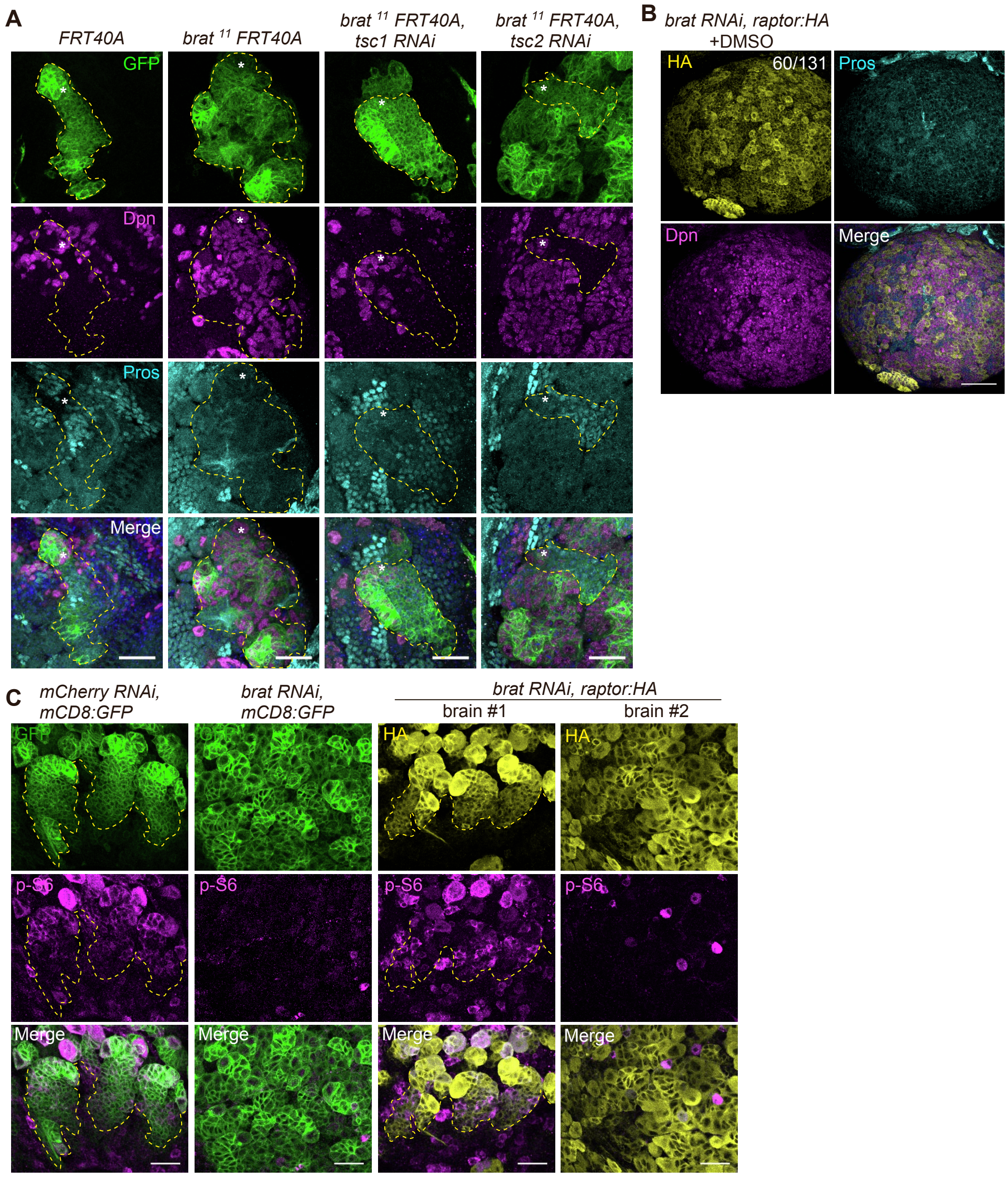

Supplementary figure 5. Driving Tor activity can rescue the Brat-depletion phenotype to restore differentiation. (Related to Figure 7) (A) Representative confocal images of MARCM clonal analysis of control (*FRT40A*), *brat* mutant (*brat*^{11} *FRT40A*), and *brat* mutant with *tsc1* or *tsc2* KD (*brat*^{11} *FRT40A, tsc1 RNAi* or *tsc2 RNAi*). Samples were stained with GFP (green), Dpn (magenta), Pros (cyan), and DAPI (blue). Dashed lines outline the clonal region. Asterisks indicate type II NBs within each clone. **(B)** Representative brain lobes of *brat* KD together with *raptor* overexpression (o/e) (*brat RNAi, raptor:HA*) flies (driven by *insc-GAL4*). Samples were stained with HA (yellow), Dpn (magenta), and Pros (cyan). The numbers

in the upper panel indicate the penetrance of the phenotype. **(C)** Representative confocal images of type II NB lineages of control (*mCherry RNAi*, *mCD8:* GFP), *brat* KD (*brat RNAi, mCD8:* GFP), and *brat* KD with *raptor* o/e (*brat RNAi, raptor:HA*). Samples were stained with GFP (green) or HA (yellow) and p-S6 (Tor activity, magenta). Dashed lines depict the border of *insc-GAL4* active regions (marked by GFP/HA). Scale bars, 50 μm (B) or 20 μm (C, A).

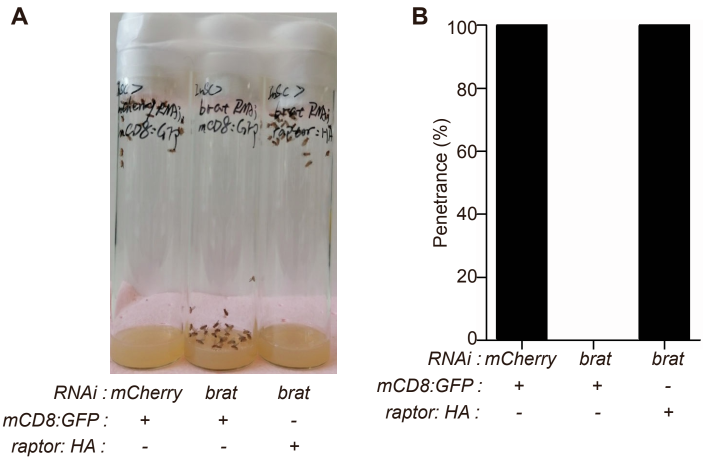

Supplementary figure 6. In a climbing assay Tor activation rescues the Brat-depletion mobility phenotype. (Related to Figure 7 and Supplementary data video 1) (A) Representative images of control (*mCherry RNAi, mCD8:GFP*), *brat* KD (*brat RNAi, mCD8:GFP*), and *brat* KD with *raptor* overexpression (o/e) (*brat RNAi, raptor:HA*) adult flies (driven by *insc-GAL4*). **(B)** Distribution of flies reaching the top of the vial in 20 seconds after knocking for controls (*mCherry RNAi, mCD8:GFP*), *brat* KD (*brat RNAi, mCD8:GFP*), and *brat* KD with *raptor* o/e (*brat RNAi, raptor:HA*).

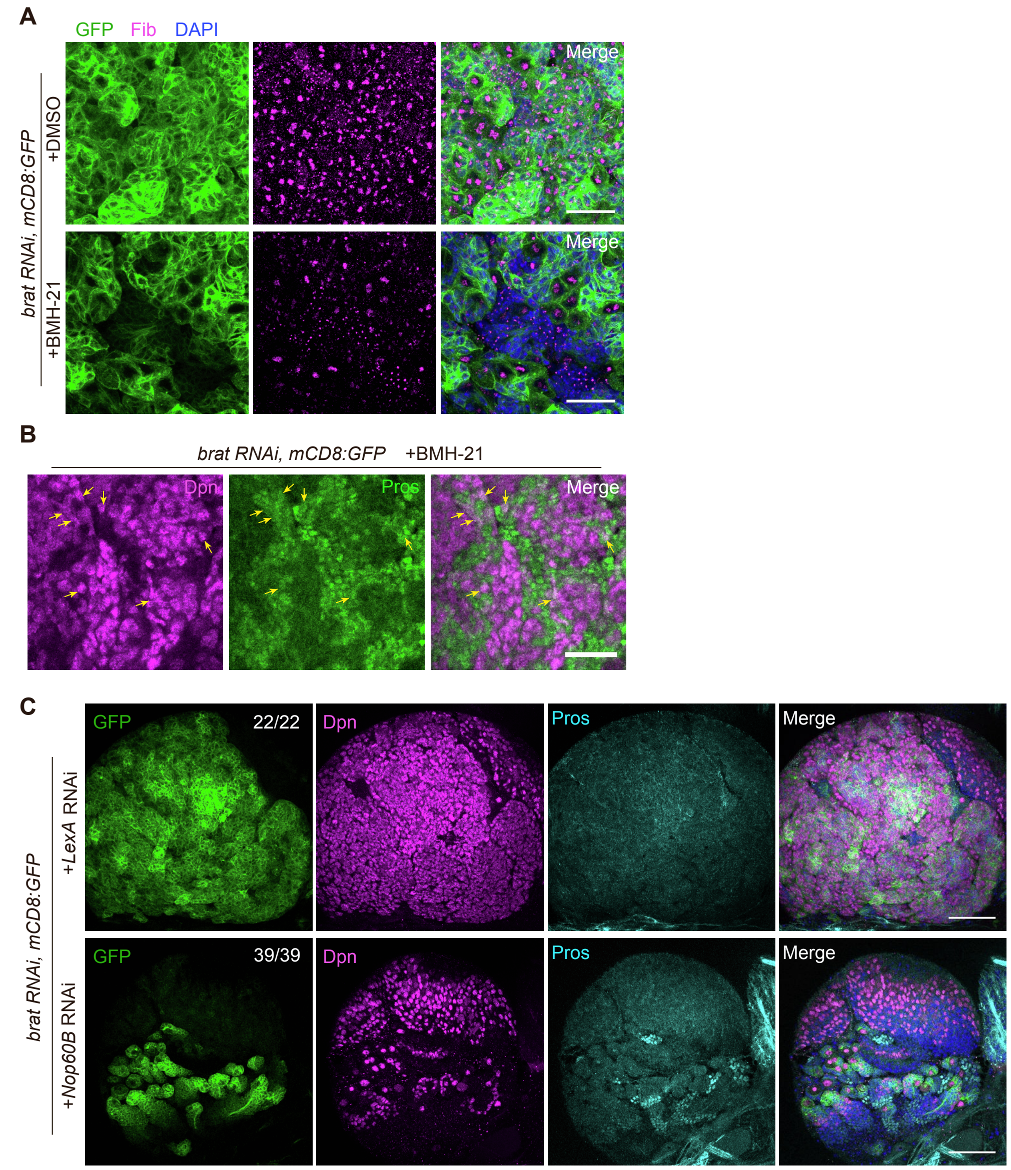

Supplementary figure 7. Inhibiting RiBi can rescue the Brat-depletion phenotype to restore differentiation. (Related to Figure 7) (A) Representative confocal images of *brat* KD (*brat RNAi, mCD8:GFP*) flies (driven by *insc-GAL4*) with or without BMH-21 feeding, stained with GFP (green), Fibrillarin (nucleoli, magenta), and DAPI (blue). **(B)** Representative confocal images of *brat* KD (*brat RNAi, mCD8:GFP*) flies (driven by *insc-GAL4*) with BMH-21 feeding, marked by Pros (green) and Dpn (magenta). Yellow arrows indicate the cells co-expressing Dpn and Pros markers. **(C)** Representative brain lobes of *brat* KD together with *LexA* RNAi or *nop60B* RNAi flies (driven by *insc-GAL4, UAS-mCD8:GFP*). Samples were

stained with Dpn (magenta), Pros (cyan), and DAPI (blue). The numbers indicate the penetrance of the phenotype. Scale bars, 50 µm (C), 20 µm (A) or 10 µm (B).

Supplementary table 1. RNA-seq reveals that RiBi related genes are downregulated by Mei-P26. (Related to Supplementary figure 2) Results of RNA-seq analysis using ovaries of control (nos-gal4/+), mei-P26 KD (RNAi, nos-gal4/UAS-mei-P26 RNAi), and mei-P26 o/e (nos-gal4/UASp-mei-P26) in a $bam^{\Delta 86}$/ $bam^{\Delta 86}$ (GSC-like) background. Table shows the log2 fold change as compared to the $bam^{\Delta 86}$/ $bam^{\Delta 86}$ background for ribosomal and ribosome biogenesis genes. RNA-seq data and fold changes for all genes has been deposited at GEO (GSE218205).

Supplementary data-video 1. Activation of Tor rescues the mobility phenotype in Brat-depleted flies. (Related to Supplementary figure 6) Representative movie of adult climbing assay. From left to right: *mCherry* RNAi control, *brat* RNAi and rescue *brat* RNAi with *UAS-raptor:HA*.
